# StimulHeat: a low-energy wearable thermal feedback device using Peltier elements with heat flow controlled loop for hand interactions in virtual reality

**DOI:** 10.1016/j.ohx.2026.e00826

**Published:** 2026-08-09

**Authors:** Matthieu Mesnage, Sophie Villenave, Bertrand Massot, Matthieu Blanchard, Pierre Raimbaud, Guillaume Lavoué, Claudine Gehin

**Affiliations:** a INSA Lyon, Ecole Centrale de Lyon, CNRS, Université Claude Bernard Lyon, CPE Lyon, INL, UMR5270, 69100 Villeurbanne, France; b Ecole Centrale de Lyon, CNRS, INSA Lyon, Université Claude Bernard Lyon 1, LIRIS, UMR5025, ENISE, 42023 Saint Etienne, France

**Keywords:** Thermal feedback, Haptics, Non-intrusive, Virtual reality, Thermoelectric effect, Low-power

## Abstract

Nowadays, the majority of wearable thermal feedback systems designed for use in virtual reality applications are not compatible or not integrated to standard controllers and are based on temperature control. The objectives of the present work are to enable integration with existing controllers, in this case Valve Index controllers, and to propose an alternative approach to managing thermal stimulation with Peltier modules by controlling heat flow instead of temperature. We introduce StimulHeat as a wireless, low power thermal feedback system, based on the continuous relationship between heat and current injection in thermoelectric device (TED). First, we designed an optimized TED driver capable of injecting a continuous, bidirectional current into the TED, thereby driving it as a heater or cooler. Subsequently, this driver was implemented in an electronic board to include temperature and heat flow control loops, as well as Bluetooth Low Energy interface for remote control. A mechanical integration was conducted, in the form of a controller extension which is non-intrusive and can be clipped to Valve Index controllers to enclose the TED, temperature sensors and electronics. Finally, we present a supplementary user study validating StimulHeat for use in Virtual Reality for short-duration thermal contact sensation, utilizing a Unity-built virtual environment with our open-source package.

## Highlights

• Robust and precise hardware for local thermal feedback via controlled heat flow.
• Wireless, non-intrusive hardware clip-on for Valve Index; adaptable to VR controllers.
• Low-power hardware driven by a custom continuous direct current source.
• Hardware with dual control loops: Heat Flow (W) or Temperature (°C) modes.
• Hardware validation shows heat flow control is faster, precise, and more comfortable.

## Graphical abstract

**Figure:**
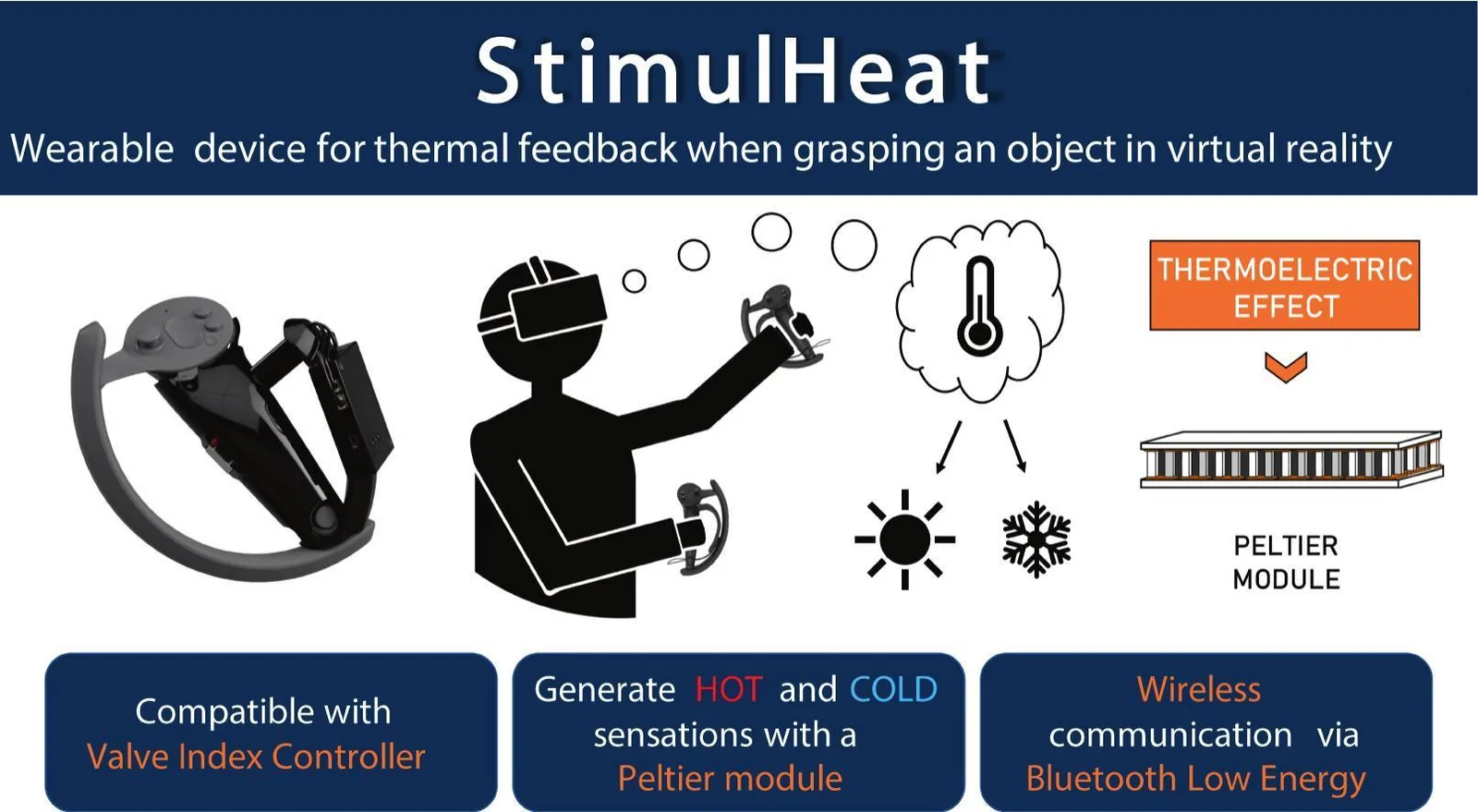

**Specifications table**

**Table:** 

Hardware name	StimulHeat
Subject area	• Educational tools and open-source alternatives to existing infrastructure
Hardware type	• Other: Haptic System for Thermal Perception in Virtual Reality
Closest commercial analog	TouchDIVER Pro gloves from WEART
Open-source license	Hardware: CERN-OHL-S v2 Firmware and Software: GPLv3
Cost of hardware	≃ 400$
Source file repository	Design files uploaded on OSF http://doi.org/10.17605/OSF.IO/HM6Q4

## 1 Hardware in context

In recent years, there has been considerable interest in the potential of thermal feedback for a wide range of future applications in the field of virtual reality (VR) [1]. From entertainment [2] to education [3], the sensation of heat or cold occupies a central position. Two options exist for providing thermal feedback: global and local stimulations [4]. The global approach is more straightforward, as it does not require a compromise between the device’s size and performance [5]. Global feedback is used to generate an ambient thermal perception, for instance by means of fans and heat lamps, as in the system developed by Villenave et al. [6]. In contrast, local feedback is used to create a sensation localized to a specific part of the body. The principal challenge for local thermal feedback is to combine miniaturization of the device with efficient heat transfer. In recent literature, several solutions have been proposed for local thermal feedback. A few research teams have investigated chemically induced thermal stimulation. For instance, Jiang et al.'s Douleur prototype [7] uses capsaicin to create a warming sensation, whereas Hamazaki et al.'s ALCool device [8] uses alcohol to induce a cooling sensation. A limited number of research teams have also explored pneumatic glove-based solutions capable of generating pressure sensations in addition to thermal feedback, such as the ThermAirGlove developed by Cai et al. [9]. However, the vast majority of proposed systems rely on thermoelectric devices (TEDs). In 2017, Ranasinghe et al. introduced Ambiotherm, which integrates several TEDs into a virtual reality headset to generate localized thermal sensations [10]. In 2020, Kim et al. employed an innovative flexible Peltier technology to develop a wearable thermal display glove [11]. More recently, in 2024, both Mazursky et al. [12] and Kang et al. [13] also utilized TEDs to provide thermal feedback. The increasing number of systems adopting this technology reflects the growing interest in thermoelectric devices for wearable thermal haptic interfaces [1].

As raised in the literature, the most common technology is thermoelectric excitation based on Peltier elements, which can produce either heating or cooling with a small device size. However, existing devices using Peltier elements exhibit certain limitations. One of the main limitations is their high-power consumption due to voltage modulation, as discussed in Section 2.3. Another limitation is that they only rely on temperature setpoints combined with Proportional-Integral-Derivative (PID) controller to adjust the intensity of the stimulation. ThermalGrasp by Mazursky et al. maintains only two temperature setpoints, 20 °C and 40 °C, using a PID controller [12]. Similarly, Flip-Pelt by Kang et al. employs a PID controller to prevent the temperature from exceeding the maximum allowable heating temperature [13]. A further limitation is the difficulty of reproducing these devices. This may be due to the limited availability of key components, such as the flexible thermoelectric device (FTED) developed by Kim et al. for their thermal display glove [11], or to the high integration density of the electronic components, combined with insufficient system documentation and the absence of the microcontroller source code, as is the case for instance for Flip-Pelt developed by Kang et al. [13]. Finally, these existing systems may be perceived highly intrusive by the user. For instance, ThermalGrasp by Mazursky et al. requires the user to wear a backpack containing the system battery [12]. Similarly, Flip-Pelt by Kang et al. requires the user to wear an external control board connected by multiple exposed wires, which may alter the natural center of gravity of the forearm and reduce wearing comfort [13]. In this context, we propose Stimulheat, a wireless local thermal feedback system based on Peltier elements. In contrast to most existing work, our device is based on heat flow control (rather than temperature). Heat flow has a direct and continuous relationship with the injected current, thus this approach eliminates the need for calibrating a PID controller, thereby increasing robustness. Moreover, the use of a Direct Current (DC) source, rather than a pulse width modulation (PWM) voltage source associated with a PID, reduces power consumption and increases the precision of the thermal response. Ultimately, the integration with Valve Index controllers [26] results in high usability and comfort. To enable the use of the device across a wide range of applications, the conventional temperature control method has also been incorporated in addition to a heat flow-based control approach. This allows users to select the most appropriate method based on their specific objectives.

## 2 Hardware description

StimulHeat is a palm-located heater/cooler system with a non-intrusive, low-power, dual control loop that is fully compatible with any virtual reality project utilising the Valve Index controllers. The selection of this particular controller was primarily determined by its forced, uninterrupted contact with the palm of the user, thereby ensuring constant thermal feedback on this contact surface. The thermoregulatory process is based on a system which incorporates a Peltier element, whereby the temperature of each of the two faces is continuously measured. Two selectable control loops have been implemented to either control heat flow or temperature. In both cases, the thermoelectric device (TED) is driven by a custom-made current source and a dedicated electronic control board, which are integrated together in a 3D-printed model designed to be easily clipped onto the aforementioned controllers. StimulHeat’s compact and wireless design allows for future integration with a diverse range of VR controllers, e.g., Meta Quest 3 [27] and Sony Playstation VR2 [28], or tangible interfaces for Augmented and Mixed Reality.

### 2.1 Custom-made current source characteristics

In order to drive a Peltier module, it is necessary to modulate the value of the current flowing through it. Two methods exist for achieving this: the direct injection of a continuous current, or the intermittent imposition of a voltage which induces an average current in turn. A review of recent literature revealed that the majority of existent systems which employ TED in VR for thermal feedback are controlled by a driver based on voltage modulation, comprising an H-bridge and utilising the PWM method to modulate the value of the voltage. Ranasinghe et al. [10] adopted in 2017 an unconventional approach by using an MC33926 motor driver, which integrates an H-bridge, to control the TED located on the neck. Similarly, Kim et al. [11] in 2020 and Mazursky et al. [12] in 2024 employed motor drivers incorporating H-bridge circuits, namely the TB6612FNG and VNH3SP30, respectively, to control their Peltier elements. Kang et al. [13] followed the same approach by using an H-bridge DC motor driver to manage their Peltier elements, although the specific driver model was not reported. Additionally, only a limited number of studies have employed a low-pass filter to smooth the output of the PWM stage, thereby converting the PWM signal into a continuously adjustable voltage source, as proposed by Watkins et al. in 2025 in their arXiv preprint [14].

**Fig. 1:**
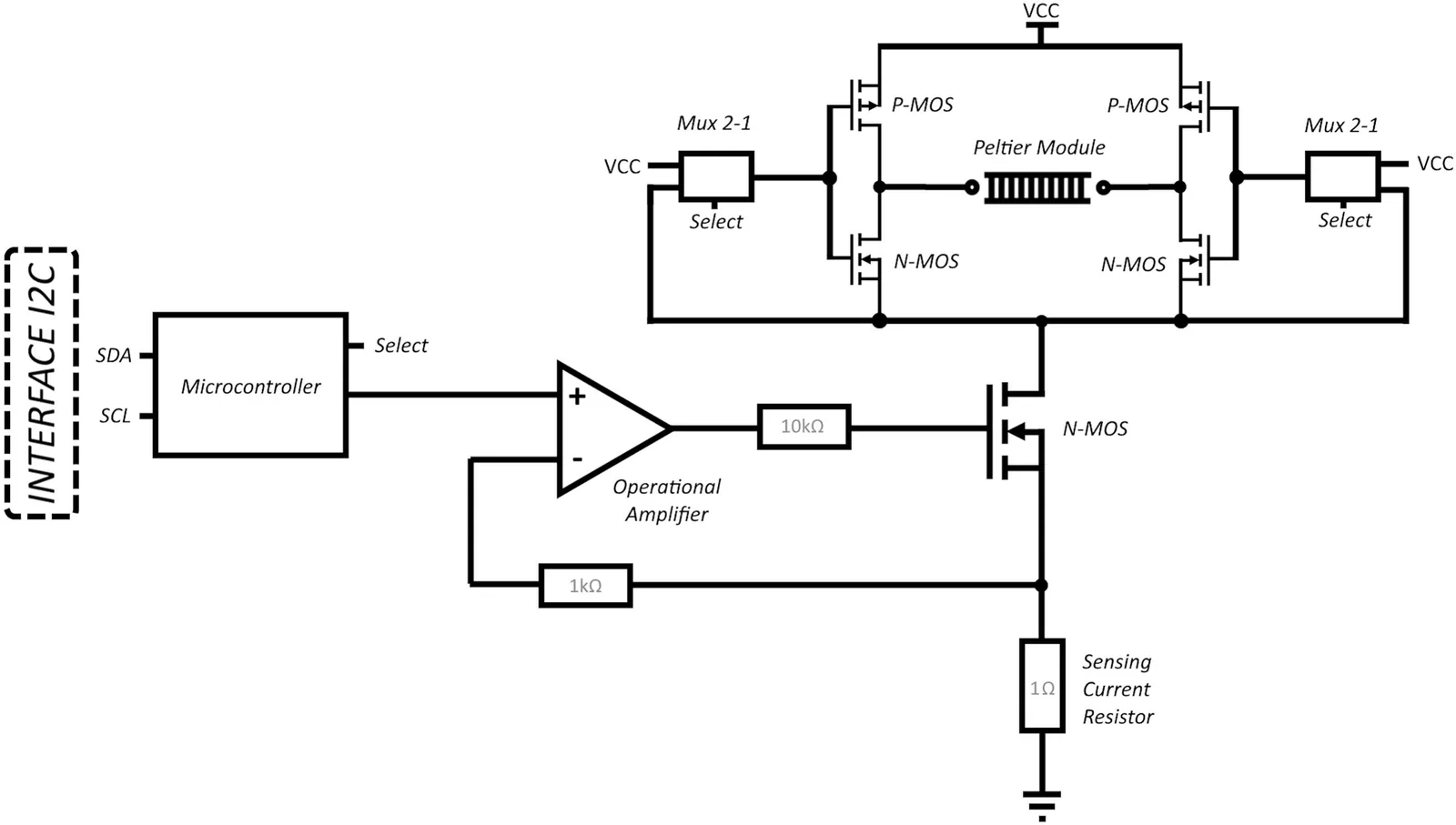
Electronic Design of driver based on continuous current source for a TED.

A study conducted by Texas Instruments [15] indicates that it is advisable to regulate a Peltier module with a continuous source of current rather than a PWM current generator to enhance efficiency, as a current control is better suited to the intrinsic behaviour of a Peltier element. Consequently, we chose to drive our Peltier module with a driver entirely based on continuous current source without a PWM stage. The circuit diagram is shown in Fig. 1. This choice of power supply benefits from the natural relationship of a Peltier element between the current injected and its ability to heat or cool through thermal conduction [16].

A microcontroller (PIC16F17115) is employed to manage I2C communication, receiving the desired output current value within the range of - 600 mA to 600 mA. This range was selected for safety reasons. The command is encoded using eight bits: seven bits represent the magnitude of the desired output current, while the eighth bit specifies the polarity (0 for positive, 1 for negative).

The microcontroller processes this numerical value by converting the seven magnitude bits into a DC voltage ($V_{\mathrm{DAC}}$) using an 8 bits digital-to-analog converter (DAC). The DAC output is fed into the non-inverting input ($V_{+}$) of an operational amplifier (LM358B). Through a closed-loop feedback circuit and the use of an N-channel MOSFET (Si4838BDY), the operational amplifier maintains a constant voltage across a current-sensing resistor equal to the DAC output voltage. Furthermore, $V_{\mathrm{DAC}}$ is limited to 0.6 V in order to prevent the current through the sensing resistor from exceeding 600 mA. Given the DAC precision, the current increment is approximately 10 mA per command step, which is sufficiently fine to remain imperceptible to human thermal skin perception. Concerning the N-channel MOSFET, it was selected for its low gate-source voltage ($V_{\mathrm{GS}}$), as a lower $V_{\mathrm{GS}}$ enables a greater current variation in response to the voltage delivered by the microcontroller unit (MCU). This configuration inherently produces a unidirectional current.

To enable both heating and cooling via conduction using a Peltier element, the current polarity must be reversible when it is injected in the Peltier element. To achieve this, the eighth bit, which encodes the polarity information of the command received via I2C communication, is sent to a select pin of the MCU. This signal controls an H-bridge circuit (comprising Si7540DP and 74LVC1G3157GW125), which surrounds the Peltier element. By reversing the current direction through the Peltier element, the H-bridge allows the system to alternate between heating and cooling operations (see Fig. 1).

Consequently, the magnitude of the current through the sensing resistor, and thus through the Peltier module, is determined by equation (1), where:

- $V_{\mathrm{DAC}}$ is the DAC output voltage applied across to the custom-made current source (in Volt)
- $I_{\mathrm{Peltier}}$ the current flowing through the Peltier module (in Ampere)

(1) $$I_{\mathrm{Peltier}}=\frac{V_{\mathrm{DAC}}}{\mathrm{SensingCurrentResistor}}=\frac{V_{\mathrm{DAC}}}{1\Omega }$$

The polarity of the current is determined by the status of the select pin: 0 corresponds to a positive current, while 1 corresponds to a negative current. In terms of voltage, this circuit drives the Peltier module to its maximum operating point, up to $VCC\;-\;V_{\mathrm{DAC}}$. This results in a maximum electrical power consumption for the Peltier element of:

$$\max\left(P_{\mathrm{peltier}}\right)=\max\left(I_{\mathrm{peltier}}\right)∙\max\left(V_{\mathrm{peltier}}\right)=\max\left(I_{\mathrm{peltier}}\right)∙\left(VCC\;-\;V_{\mathrm{DAC}}\right)=0,6∙\left(3,7\;-\;0,6\right)=1.86W$$

### 2.2 Heat flow or temperature control modes

**Fig. 2:**
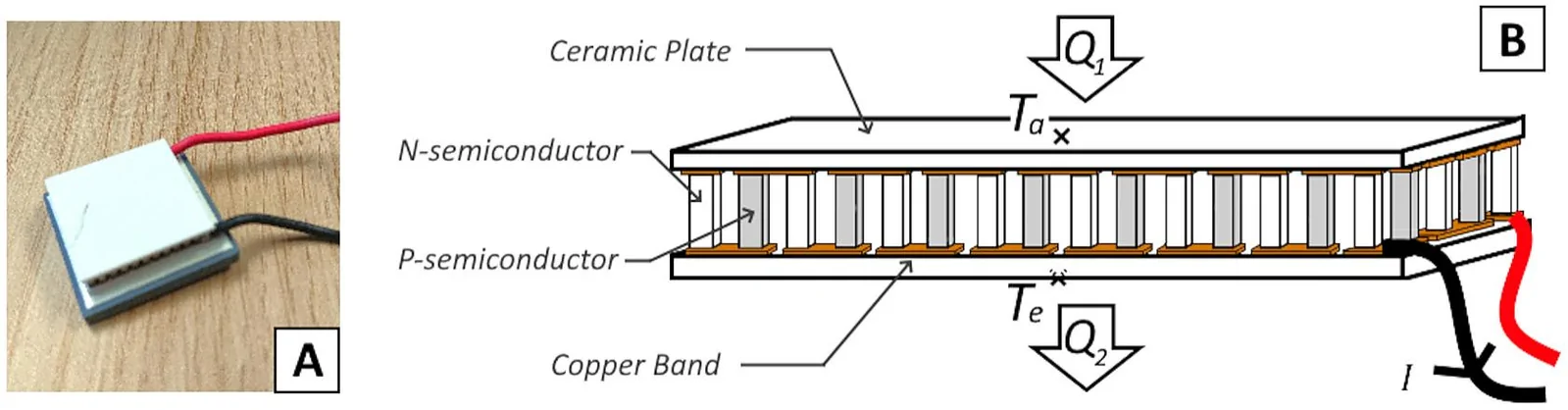
A: Picture of a TED with a ceramic heat sink − B: Schematic of a TED.

Stimulheat incorporates two methods of controlling the Peltier element, as the device is powered by a voltage-controlled, reversible, direct current source. Based on the theoretical laws and the operating diagram of a Peltier module shown on Fig. 2, it can be demonstrated that the heat flow passing through it is given by equation (2), where:

- $Q_{1}$: Heat flow which occur on the absorbed side of the Peltier Module (in Watt, or joules per second)
- R: Electrical resistance equivalent of the Peltier module (in Ohm)
- α: Seebeck coefficient equivalent of the Peltier module (in Volt per Kelvin)
- $\theta_{m}$: Thermal resistivity equivalent of the Peltier module (in Kelvin per Watt)
- $T_{a}$: Temperature of the absorbed side (in Kelvin)
- $T_{e}$: Temperature of the emitted side (in Kelvin)
- I: Continuous current injected (in Ampere)

(2) $$Q_{1}=-\frac{R}{2}∙I^{2}+\alpha ∙T_{a}∙I+\frac{T_{a}-T_{e}}{\theta_{m}}$$

From this standpoint, two distinct approaches to control stand out. The initial approach involves the direct management of the value of the heat flow. Based on the second-order equation (2), it is possible to control a given heat flow by injecting a specific current value, and conversely, to determine the required current from a desired heat flow. By positioning the Peltier module in direct contact with the skin, the primary mechanism of heat transfer is facilitated through conduction. The loss of heat due to convection is minimised by ensuring effective thermal contact between the Peltier module and the skin. The second method involves the injection of current in accordance with the difference between a temperature setpoint and the temperature measured at the contact zone between the TED and the skin. The incorporation of a Proportionnal-Integral-Derivative (PID) controller serves to improve the precision and response time of the targeted temperature. The default values of proportional gain (Kp = 300), integral gain (Ki = 50) and derivative gain (Kd = 20) of the PID controller are embedded in the firmware; however, these parameters can be adjusted through the wireless interface. The initial default parameter values were determined using the Ziegler–Nichols method. The selection of each control methods depends on the specific requirements of the VR application, directly tied to the design of the interactive graspable object within the VE. In circumstances where the object is characterised as a continuous heat source, the utilisation of a heat flow control may be recommended. Conversely, in scenarios where the grasped object, once grasped, tends to approach a temperature close to the body’s skin temperature over time, a temperature control may be more appropriate. A technical characterisation of both control methods is presented in *Validation and characterization* section.

We hypothesise that heat flow control is more appropriate for thermal perception in cases where there is direct contact between an object and the skin and particularly when using a Peltier element. First, from an energy efficiency standpoint, it is more effective to directly control heat flow rather than surface temperature Ta (see Equation (2)). Second, when a real object is grasped and perceived, the sensation is not solely determined by temperature. Several studies have shown that even a nearly constant surface temperature can still produce distinct thermal sensations [17], [18]. This phenomenon is attributed to the thermal effusivity of the object’s material [19], [20]. This can be illustrated by a common observation: in a room maintained at a constant temperature of 20 °C, a wooden spoon typically feels warmer than a metal one, despite both being at the same initial temperature. This occurs because steel (a metal) transfers heat more effectively to an external body than wood does—provided that the body it contacts has a lower thermal effusivity. Consequently, the rate of temperature change during contact, until a steady-state level is attained, is higher for the steel spoon than for the wooden one. Therefore, to reproduce a realistic thermal sensation on the skin using a single material with a Peltier element, it may be more appropriate to modulate the heat flow rather than to fix the surface temperature. Using PID controller to maintain a specific temperature would impose a fixed rate of temperature change, which may not be suitable for replicating the sensation associated with materials of different thermal effusivities.

To summarize, the device can be controlled in two different ways: by using a heat flow setpoint (in Watts) or a temperature setpoint (in Celsius). These utilisations of the wireless protocol implemented over Bluetooth Low Energy, using Protocol Buffers, allows for two distinct options. The user can select a generic setpoint from VERY HOT to VERY COLD, with five predefined levels for both heat and temperature: for heat, VERY HOT (- 4 W), HOT (- 2 W), NEUTRAL (0 W), COLD (2 W), and VERY COLD (4 W); for temperature, VERY HOT (41 °C), HOT (38 °C), NEUTRAL (35 °C), COLD (32 °C), and VERY COLD (29 °C). Alternatively, a specific setpoint value can be defined, within the heat range of [- 9 W; 9 W] and the temperature range of [15 °C; 42 °C]. The aforementioned ranges have been deduced from the capacity of heating and cooling of our TED (ET-071–08-15) for the heat range, and from the temperature range that does not affect the nociceptors [21], [22] for the temperature range. In principle, both ranges are accessible; in practice, positive values of cold heat flow (>6 W) and cold temperatures (<25 °C) would require the addition of a high-performance heat sink system to effectively dissipate the heat produced on the opposite side of the TED when it is used for cooling.

### 2.3 Low-power solution

**Fig. 3:**
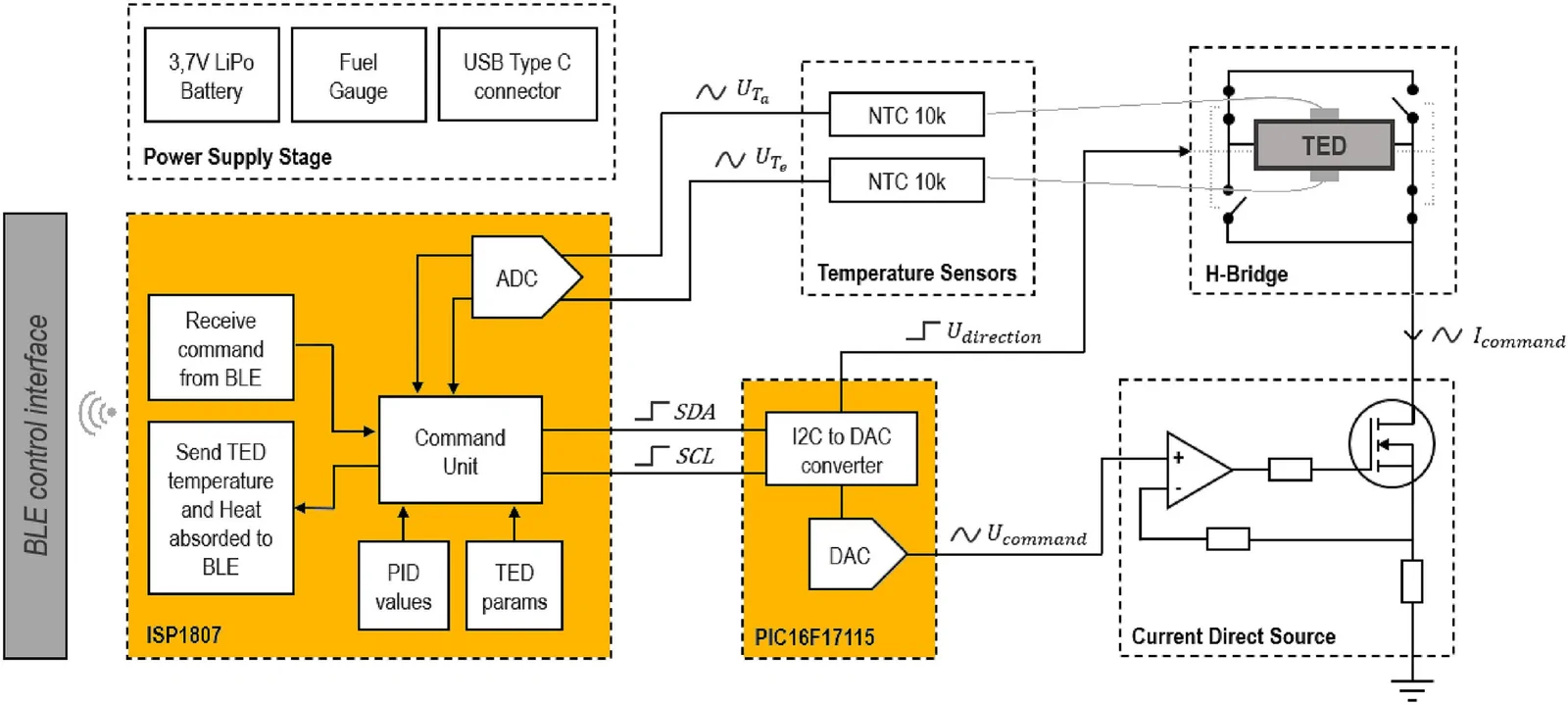
Flowchart of Stimulheat from the BLE command received to the current control through the Peltier.

**Fig. 4:**
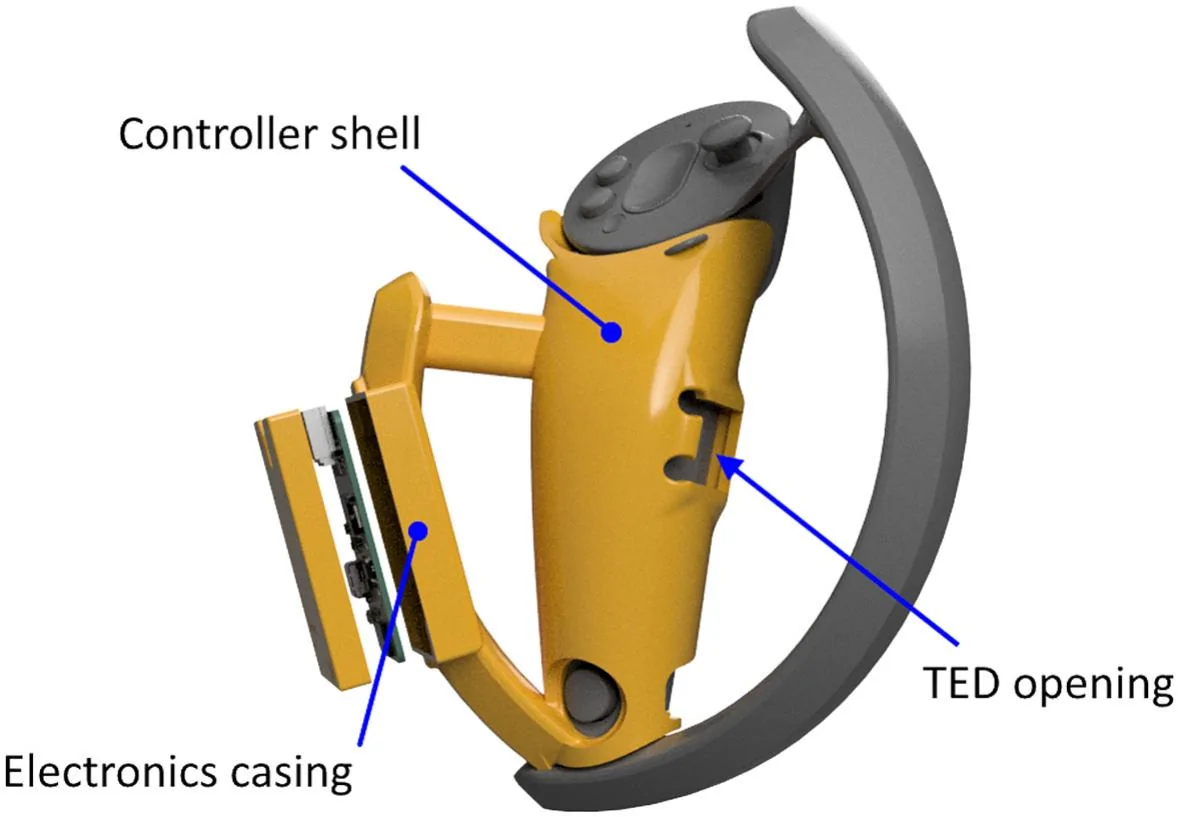
3D model of the accessory fitting on the Valve Index controller.

StimulHeat is a low-power solution that employs a low-power communication protocol and a high-energy efficiency method of TED’s control, making it integrable in a handheld device such as a VR controller, as shown in the flowchart in Fig. 3. It can be used without the need for an external battery carried by the user on a distinct body site (such as the forearm, the wrist, or in a backpack), in contrast to other existing implementations: for instance, Mazursky et al. required users to wear the external battery in a backpack [12], whereas Kang et al. used a relatively heavy battery attached near the user's elbow [13]. The PCB and the external battery are housed within a small enclosure on a 3D printed shell clipsable on the Valve Index controller (shown in Fig. 4). In order to facilitate remote control, an ISP1807 microcontroller (from Insight SIP), which is a nRF52804-based system-in-package (Nordic Semiconductors), was utilised for its proven low current consumption and its integrated Bluetooth Low Energy (BLE) interface, which significantly reduces energy consumption whilst ensuring interoperability and reliable command reception. Its role is to manage the control of the current injected into the Peltier element according to the temperature measured by the thermistors (see Fig. 3). It sends commands over the I2C bus to a dedicated Peltier element driver through an interface based on the PIC16F17115 microcontroller (from Microchip Technology), which, as described previously in the section “2.1. Custom-made current source characteristics,” converts these digital commands into analog control signals that regulate the magnitude and the polarity of the injected current. As shown in orange in the Fig. 3, two different microcontrollers are used for two main reasons. First, the ISP1807 does not integrate a digital-to-analog converter (DAC). Second, the PIC16F17115 enables precise control of the current injected into a single TED while maintaining very low power consumption (only a few milliwatts). This design choice also facilitates future scalability, as additional TED drivers can easily be added by connecting them to the same I2C bus.

Regarding the selection of the battery, we installed a small rechargeable LiPo battery (L.l.h: 45 mm x 34.5 mm x 6.5 mm) of 3.7 V with a capacity of 850 mAh. It was determined that the application of a current of less than 600 mA on the ET-071–08-15 series (TED from European Thermodynamics), previously selected based on three specific parameters (α, θm, R), resulted in a pronounced thermal sensation, both in the cold and in the heat (under the condition that the current is inverted in the latter case). In addition, the TED functions at a voltage of 3.7 V, which results in one StimulHeat’s maximum power consumption of 2.22 W, and 4.44 W for both controllers. For comparison with existing devices that provide thermal feedback to the hand or other body areas using TEDs, the Ambiotherm system from Ranasinghe et al. consumes approximately 13 W when using two Peltier elements for thermal stimulation at the neck [10]. The ThermalGrasp system from Mazursky et al. operates at around 25 W using a Peltier-based setup [12]. The Flip-Pelt device from Kang et al. consumes up to 9.6 W by using eight TEDs on the forearm [13], while the thermal display glove from Kim et al. consumes an average of 1.8 W using four custom-made flexible TEDs [11].

### 2.4 Integration on Valve Index controllers

We designed an integration module for the Valve Index controllers. Initially, the STL file provided by Valve Corporation is utilised (Github), as it is designed to be an accessory shell that can be added to a Valve Index controller. This was originally created with the intention of accommodating individuals with longer thumbs or to be used as a base for additional accessories. Fig. 4 illustrates the 3D printed custom controller shell that we created from the previous STL file. A square opening has been engineered to receive the Peltier element, situated at palm level. An extension of the controller has been incorporated to encapsulate the electronics without compromising the user’s freedom of movement. Two grooves with tubes have been embedded within the 3D model to facilitate wire routing from the housing to the Peltier element.

This integration enables any user of Valve Index controllers to utilise a haptic thermal feedback system when grasping an object in a VE. For the article purpose, the integration will be demonstrated solely on the Valve Index controllers, as the innovation lies predominantly in the StimulHeat device itself. Nevertheless, this integration can readily be adapted to other controllers, such as Meta Quest Touch [29] or Vive Controllers [30], for example.

In summary, by reproducing StimulHeat you will have access to:

• A low-cost, bidirectional current source specifically designed to efficiently drive thermoelectric devices (TEDs) enabling both positive and negative heat flows for hot and cold thermal stimulation.
• A scalable I^2^C-based control architecture that can be extended to support multiple independent thermal stimulation channels.
• A dual control framework combining temperature and analytical heat flow regulation for precise thermal stimulation with TEDs.
• An easy-to-use implementation of a thermal display for VR applications integrated in Valve Index controllers
• An open-source and reproducible platform that can be adapted to applications beyond VR, including thermal haptics, psychophysics, and biomedical research.

Furthermore, StimulHeat was showcased in the research demonstration track at the IEEE VR 2025 conference [23], revealing significant interest from the VR community in such a device.

## 3 Design files summary

As shown in Table 1, all design files are released under open-source licenses. Additional details pertaining to each design file are provided below.

• StimulHeat-3D-Parts: ZIP File which contains the printable 3D models in STEP and STL formats (for SLA or FDM).
• StimulHeat-Hardware: ZIP File which contains the entire KiCAD project, including the schematic (stimulheat.kicad_sch file), the PCB (stimulheat.kicad_pcb file), the symbol library (stimulheat.kicad_sym file), the footprint library (stimulheat.pretty folder) and the 3D model of the components library (stimulheat- 3dmodels folder). Also manufacturing (GERBER and DRILL) files, assembly data (component position placing file, assembly drawing, bill of materials) are provided for a complete manufacturing and assembly of the printed circuit board.
• StimulHeat-Firmware-PIC16F17115: ZIP File which contains all the source code to program the PIC16F17115 on the StimulHeat PCB. A prebuilt binary file is also provided for direct programming of the microcontroller.
• StimulHeat-Firmware-ISP1807: ZIP File which contains all the source code needed to program the ISP1807 on the StimulHeat PCB. A prebuilt binary file is also provided for direct programming of the microcontroller.
• Stimulheat-WebApp: ZIP file which contains the local standalone HTML/JS web application allowing you to test your StimulHeat device.
• StimulHeat-Unity: ZIP file which contains the Unity virtual demo scene used in the *Validation and characterization* section.

**Table 1:** Summary of the StimulHeat design files, their corresponding open-source licenses, and file locations.

**Design file name**	**File type**	**Open-source license**	**Location of the file**
StimulHeat-3D-Parts	ZIP File (3D STEP files)	CERN-OHL-S v2	StimulHeat − 3D Parts on OSFDOI
StimulHeat-Hardware	ZIP File (ECAD files)	CERN-OHL-S v2	StimulHeat − Hardware on OSFDOI
StimulHeat-Firmware- PIC16F17115	ZIP File (C source code)	GPLv3	StimulHeat − Firmware − PIC16F17115 on OSFDOI
Stimulheat-Firmware- ISP1807	ZIP File (C source code)	GPLv3	StimulHeat − Firmware − ISP1807 on OSFDOI
StimulHeat-WebApp	ZIP File (HTML/JS files)	GPLv3	StimulHeat − WebApp on OSFDOI
StimulHeat-Unity	ZIP File (Unity project)	GPLv3	StimulHeat − Unity on OSFDOI

**Table 2:** Comprehensive bill of materials (BOM) for the StimulHeat system, summarizing each component’s.

**Designator**	**Component**	**Number**	**Cost per unit −currency**	**Total cost −** **currency**	**Source of materials**	**Material type**
PCB1	StimulHeat PCB	1	∼ 150 − $	∼ 150 − $	*Eurocircuit*	*Others*
3D1	3D Print − Main	1	∼20 − $	∼20 − $	*DIY or**JLCPCB*	*Others*
3D2	3D Print – PCB Housing	1	∼5 − $	∼5 − $	*DIY or**JLCPCB*	*Others*
3D3	3D Print – TED Support	1	∼2 − $	∼2 − $	*DIY or**JLCPCB*	*Others*
PRG1	PicKit5 PG164150	1	74.90 − $	74.90 − $	Octopart	*Others*
ADT1	Tag-Connect cable for PicKit5 TC2030-PKTNL	1	43.00 − $	43.00 − $	Tag-Connect	*Others*
PRG2	J-LINK EDU MINI 8.08.91	1	60.00 − $	60.00 − $	Octopart	*Others*
ADT2	Tag-Connect cable for ARM TC2030-CTXNL	1	42.95 − $	42.95 − $	Tag-Connect	*Others*
ADT3	Tag-Connect clips TC2030-CLIP-3PACK	1	18.00 − $	18.00 − $	Tag-Connect	*Others*
BAT1	Battery LP603443JU	1	9.17 − $	9.17 − $	Octopart	*Others*
OPT1, OPT2, OPT3	Optopipe 51513020250F	3	1.01 − $	3.03 − $	Octopart	*Others*
THR1, THR2	Thermistor GA10K3MCD1	2	13.73 − $	27.46 − $	Octopart	*Others*
TED1	TED ET-071–08-15	1	46.30 − $	46.30 − $	Octopart	*Others*
HSK1	Heat Sink MPC202025T	1	1.10 − $	1.10 − $	Octopart	*Others*
SCR1, SCR2	M2 Screws − RM2X8MM 2701	2	0.85 − $	1.70 − $	Octopart	*Others*
NUT1, NUT2	M2 Nuts − MHNZ 002 4	2	0.10 − $	0.20 − $	Octopart	*Others*
WRH1	Wiring harness - 2,162,711,060	1	2.77 − $	2.77 − $	Octopart	*Others*
HST1	Heat shrinkable Tube 1 mm RNF-3000–3/1–0-SP	≃ 1 m	3.12 − $	3.12 − $	Octopart	*Others*
HST2	Heat shrinkable Tube 0.5 mm RNF-3000- 1.5/0.5–0-SP	≃ 1 m	1.15 − $	1.15 − $	Octopart	*Others*

All components required to reproduce the StimulHeat device are listed in Table 2. Additionally, supplementary information specific to certain components is provided below.

## 4 Bill of materials summary

• Additional descriptions − Global: Please find below a list of materials that may not already be included in the previous list. These are workbench / assembly tools which can be useful to help assembling the device manually as it is described in the Build Instructions section:
  o A wire stripper to remove the insulation from electrical wires − Octopart − Price: 120,19 $
  o A hot air iron to heat the heat shrink tubing, enabling it to be retracted on itself. This, in turn, ensures electrical isolation and improves mechanical resistance. − Octopart − Price: 159.14 $
  o A soldering iron to solder component together − Octopart − Price: 51.25 $
  o A polyimide adhesive tape (or any other tape that can resist to heat) to fix the thermistors on TED sides − Octopart − Price: 2.84 $
• Additional descriptions – PCB1 − StimulHeat PCB: There are two options available to obtain an assembled PCB for the electronic board. You can either manufacture the PCB yourself or outsource its production. We are recommending the second option, given the complexity of some component packages, if you only have a soldering iron. Here is a list of subcontractors able to reproduce the project: JLCPCB, PCBWay (Worldwide), EuroCircuits, Proto-Electronics (European) and EMS FACTORY (France). In order to proceed, please provide the folder’stimulheat export’ which can be found within the’stimulheat pcb’ ZIP file (Section 5.2).
• Additional descriptions − 3D1, 3D2 and 3D3: As with the StimulHeat PCB, you can print the 3D model yourself or use an external subcontractor. For the realisation of this project, we worked with JLCPCB to print the differents 3D model using SLA technology. Other possible subcontractors are Protolabs Network, Shapeways, Protolabs, etc…
• Additional descriptions – TED1: The TED selected in the materials bill is the result of a Peltier benchmark established by our own research. It is important to note that the utilisation of an alternative product from an alternative supplier is permissible and should not result in a significant change to the performance of StimulHeat. Adapting your Web or VR applications for use with your selected TED only requires changing the αm, θm and Rm parameters accordingly to your TED specifications. However, it is recommended that a similar product is employed. The following is a concise, non-exhaustive list of Peltier elements that may be utilised as an alternative to the one that has been proposed: CP20251 − (Octopart); CP30239H (Octopart); CP20247H (Octopart); …

## 5 Build instructions

To build the project as described below, components listed in the bill of materials are required. The process consists of four main steps which have to be repeated for both left and right controllers: 1) assembling the wiring harness, which includes the TED and two thermistors; 2) installing the LiPo rechargeable battery; 3) programming the microcontrollers; and finally, 4) completing the full assembly of the system.

### 5.1 Assembly safety precautions

Before starting the assembly, ensure that the following safety precautions are observed:

• Wear appropriate personal protective equipment (PPE) throughout all soldering operations, including safety goggles to protect against molten solder splashes and heat-resistant gloves to reduce the risk of burns. Solder only in a well-ventilated area or, preferably, with a fume extractor to minimize exposure to solder fumes.
• Store the LiPo battery separately until it is ready to be connected. Never puncture, crush, bend, or expose the battery to excessive heat. Before installation, carefully inspect the battery and do not use it if it appears swollen, damaged, or leaking.
• Follow all relevant safety procedures when performing wiring, cutting, or heat-shrinking operations. Read and comply with the safety instructions provided for all tools and equipment used during assembly.
• If the LiPo battery becomes excessively hot or shows signs of swelling or smoke, disconnect it immediately and move it to a safe location if this can be done without risk.
• Wash your hands thoroughly after completing the assembly to remove any residues from soldering materials and other contaminants.

For information, the LiPo battery complies with the international standard IEC 62133 for portable sealed secondary cells and batteries, as well as the equivalent US requirements under the OSHA Hazard Communication Standard (29 CFR 1910.1200). Under normal operating conditions, the battery presents a low chemical and electrical risk due to its sealed design and low operating voltage (3.7 V).

### 5.2 PCB1 manufacturing and assembly procedure

To manufacture and assemble PCB1, the following procedure shall be followed. First, the manufacturing files must be prepared by downloading the “StimulHeat-Hardware-main/Export/” directory, which contains all the required production files, including the Gerber files (.gbr), drill files (.drl), bill of materials (BOM) file (.csv), and component placement file (.pos). These files shall then be uploaded to the selected PCB manufacturing platform. For automated assembly, the “PCB-A” assembly option shall be selected, allowing the manufacturer to perform the component mounting process. The required manufacturing parameters shall then be configured according to the following specifications:

• Base material: FR-4
• Number of layers: 2
• PCB thickness: 1.6 mm
• Surface finish: HASL
• Minimum via hole size/diameter: 0.3 mm
• Minimum track width / spacing between tracks: 0.15 mm

For in-house fabrication and manual assembly of PCB1, special attention must be given to the soldering requirements of the ISP1807 microcontroller. Due to its package characteristics, this component requires appropriate soldering equipment and techniques that may not be available with standard electronic prototyping tools. Therefore, the feasibility of manually assembling this component should be assessed beforehand to ensure a reliable fabrication process.

### 5.3 Creation of the wiring harness containing the TED and the two thermistors

**Fig. 5:**
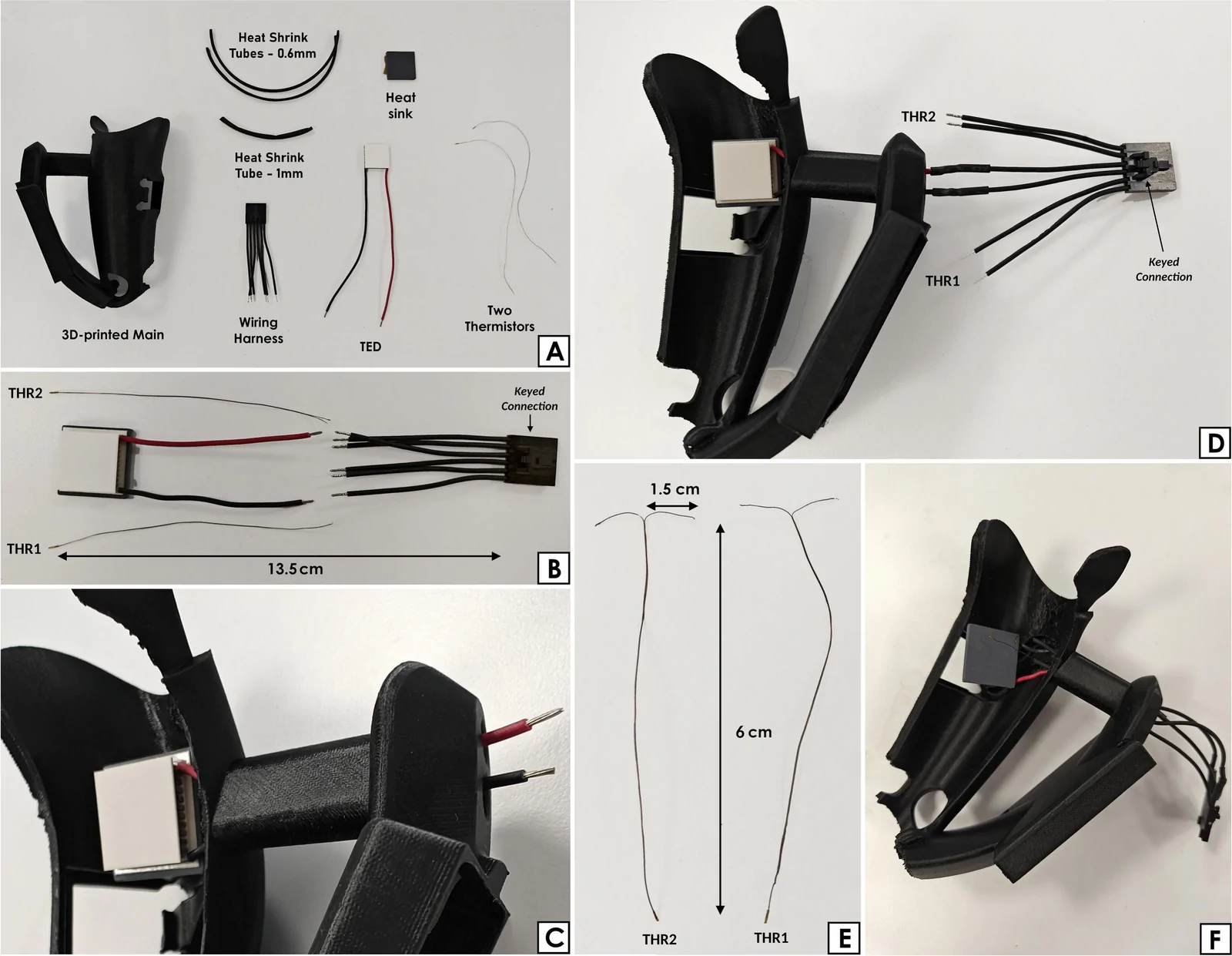
Building instructions − Wiring Harness. A − Exploded view of the required hardware components; B − Maximum overall dimensions of the wiring harness; C − Location of the tube in the 3D-printed main body for routing the TED wires, ensuring that the red wire is routed through the upper tube; D − Wire function distribution within the wiring harness; E − Recommended thermistor lead length before soldering to the wiring harness; F − Picture of the complete assembled system. (For interpretation of the references to colour in this figure legend, the reader is referred to the web version of this article.)

To complete this step, you will need the 3D print − Main (3D1), the thermistors (THR1, THR2), the TED (TED1), the heat sink (HSK1), the wiring harness (WRH1) and the heat shrink tube (HST1, HST2) as it is shown in Fig. 5 − A.

• First, mount HSK1 onto TED1, ensuring it is properly centered. Next, based on the length of TED1′s electrical wires, trim the two central wires of WRH1 so that the combined length of TED1 wires and WRH1 wires is exactly 13.5 cm. Cut THR1 and THR2 wires to a length of 7.5 cm, and trim the last four wires of WRH1 to leave 1 cm exposed on each. Finally, strip the insulation from TED1 wires using a wire stripper. The final setup should match the configuration shown in Fig. 5 − B.
• Then, cut six pieces of HST1, each 1.5 cm long. Slide two of them onto the two central wires of WRH1 and reserve the remaining four for THR1 and THR2 wires. Next, pass TED1 wires through the two holes in 3D1, as shown in Fig. 5 − C. Solder TED1 wires to the two central wires of WRH1. After soldering, cover the joints with HST1 and shrink them using a hot air gun to ensure proper electrical insulation. The resulting configuration should look like the Fig. 5 − D.
• Prepare the two thermistors THR1 and THR2 as illustrated in Fig. 5 − E, by gently separating their two wires over a length of approximately 1.5 cm. Although the insulation is already removed, handle the wires carefully as they are very thin and fragile. To reinforce them, cut four pieces of HST2, each 7 cm in length. Slide them on THR1 and THR2 wires and shrink them using a hot air gun. Strip the last four wires of WRH1, then carefully solder THR1 and THR2 wires to their corresponding wires on WRH1, as illustrated on Fig. 5 − D. Ensure that the keyed connector is oriented correctly before making the soldered connections. On one of the two solder joints per thermistor, place a piece of Kapton tape to provide additional electrical insulation. Then, slide the remaining four 1 mm thermal sheaths over the solder joints and shrink them using a hot air gun to ensure proper insulation. Verify that the two solder joints on each thermistor do not touch each other by measuring the resistance between the two THR1 wires, then between the two THR2 wires. A reading of 0 O indicates a short circuit, in which case the step must be redone.
• Once the previous step is complete, route the wires through the tubes of the 3D1, following the configuration shown in Fig. 5 − F. Finally, tape THR1 onto the face of the TED1 using Kapton tape. Ensure that THR1 is attached to the face of TED1 without HSK1. Then, tape THR2 to HSK1 using Kapton tape.

### 5.4 Installation of the rechargeable battery

For this step, you will need the StimulHeat PCB (PCB1) and the 3.7 V − 850 mAh LiPo Battery (BAT1).

**Fig. 6:**
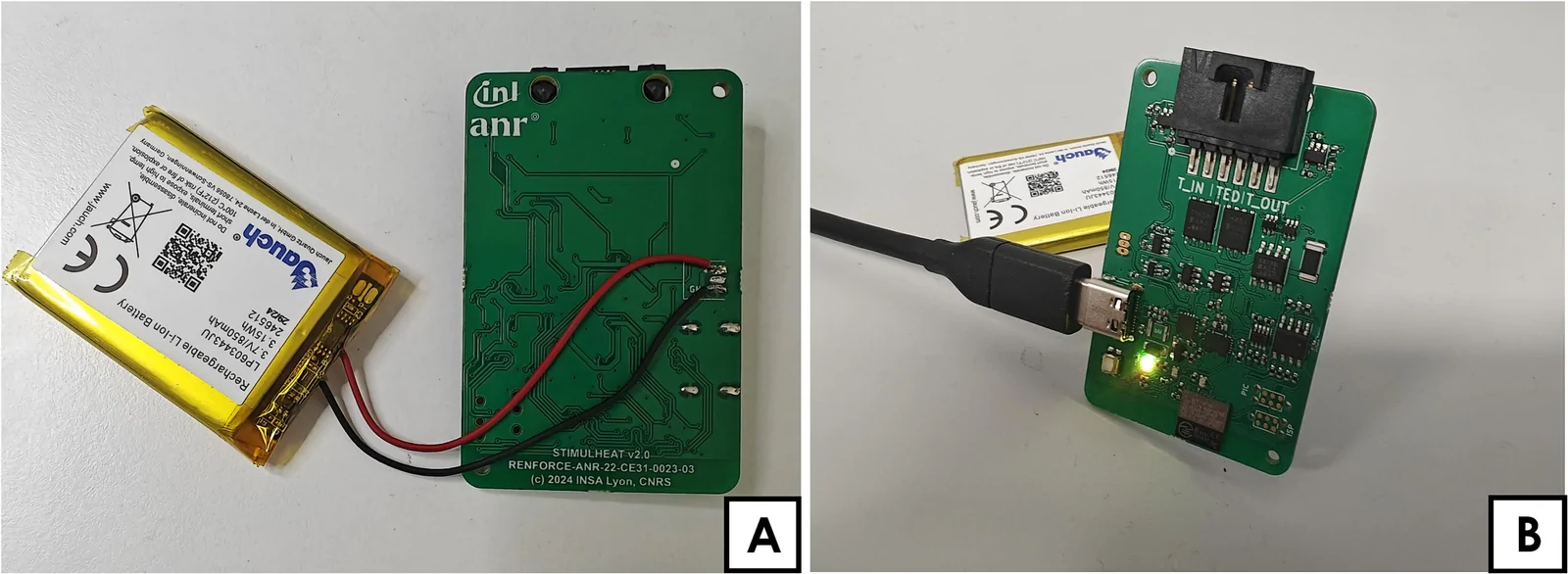
Building instructions − Rechargeabable Battery Installation. A − Soldering the battery connector to the StimulHeat PCB. Ensure that the battery's black wire is connected to the GND pad; B − Test procedure to verify that the battery connectors has been soldered correctly.
• To check if the soldering is done properly, try charging your battery with a USB-C cable. If a yellow LED light comes on, as shown in Fig. 6 − B, this means that the device is well connected to the battery and working properly. If not, check your welding. The yellow LED will turn green when the battery is fully charged.

• Using a soldering iron, solder the black wire of BAT1 to the GND pad on the bottom side of PCB1, which is one of the three pads of the battery connector. Next, solder the red wire of BAT1 to the pad marked with an arrow. Be sure to follow this connection sequence to avoid any electrical risks. At the end of this step, your assembly should look similar to the example shown in Fig. 6 − A.

### 5.5 Programming of the micro-controllers

The next step consists in programming the two microcontrollers on the PCB. You will need the previous assembly of the StimulHeat and the rechargeable battery, as well as the Pickit5 (PRG1), the Tag-Connect cable for PicKit5 (ADT1), the J-LINK EDU MINI (PRG2), the Tag-Connect cable for ARM (ADT2) and Tag-Connect Clip (ADT3).

• Start by charging your battery. The charging LED will turn yellow if the battery is not fully charged. Once the charging LED indicator turns green, programming can be done. Turn on your PCB1 using the push button.
• We begin with the microcontroller PIC16F17115 by using PRG1 and ADT1. To do so, first connect the adapter to your PRG1, ensuring the MCLR pin on the PicKit5 (indicated by an arrow) matches the blue marked wire on the ADT1. Next, connect the other end of the programming cable to the ’PIC16F’ connector on PCB1, and maintain it with ADT3. Finally, plug the USB cable into PRG1 and your computer, then open MPLAB X IPE. Load the hex file ’**stimulheat-firmwarepic16f17115.production.hex**’ (located in stimulHeat pic16f code.zip). Select All Families for Family, PIC16F17115 for device and PicKit5 for your tool. Then click on Program. At this time, the PIC16F17115 is programmed.
• Now, let’s proceed with programming the microcontroller ISP1807. For this step, you will need the PRG2 with its programming cable ADT2. Start by connecting the cable to PRG2, then connect the other end to the ’ISP1807’ connector on the PCB1 by maintaining it with ADT3. Finally, connect the USB cable to PRG2 and your computer, then open nRF Connect for Desktop. Launch it, find the”Programmer” App, install it and then open it. On it, click on’select device’ and select JLINK. By clicking on add file in File menu, select the hex file ’**zephyr.hex**’ (located in the ‘build/zephyr’ directory of the archive stimulHeat isp1807 code.zip). Then, click on’erase and write’ in Device menu. At this time, the ISP1807 is programmed.

### 5.6 Assembly of the whole project

To assemble the entire project, you will need the first sub-assembly containing the wiring harness, and the second sub-assembly composed of the StimulHeat module and the battery. You will also need the the 3D Print − PCB housing (3D2), the 3D Print − TED Support (3D3), the optopipes (OPT1, OPT2, OPT3), the M2 screws (SCR1, SCR2) and the M2 nuts (NUT1, NUT2).

**Fig. 7:**
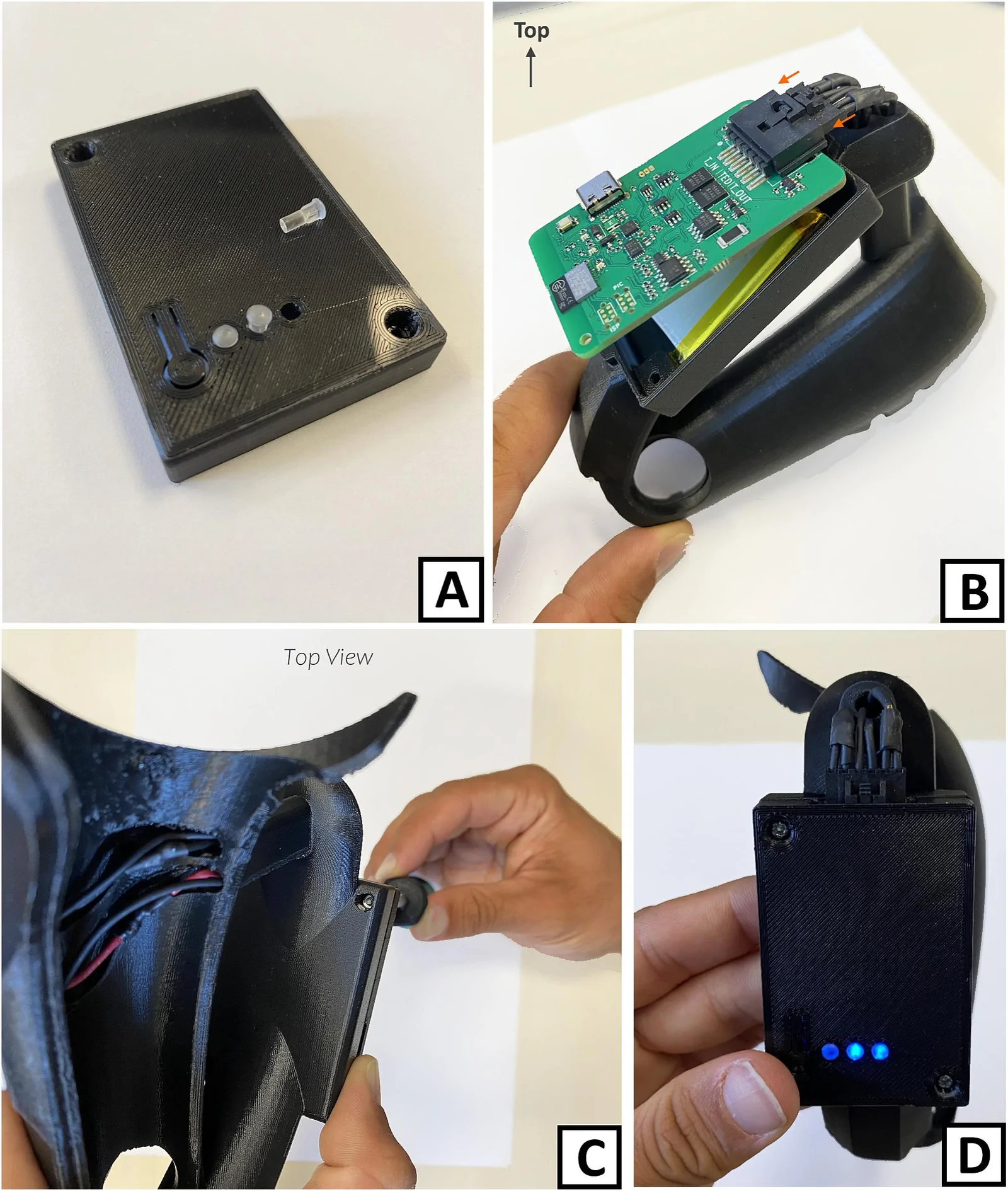
Building instructions − Assembly of the whole project. A − Assembly of OPT1, OPT2, and OPT3 onto the 3D2 component. B − Connection of the StimulHeat PCB + battery to the 3D-printed main structure using WRH1. C − Assembly of the 3D-printed PCB housing + optopipe assembly with the StimulHeat PCB + battery + 3D-printed main structure using screws and nuts. D − Test procedure to verify correct installation.
• Then, insert the StimulHeat PCB + battery assembly into the dedicated slot on the main 3D-printed structure from the first sub-assembly. To help with orientation, make sure the connector for WRH1 on the StimulHeat is positioned at the top similar to the example shown in Fig. 7 − B.
• Insert NUT1 and NUT2 into the designated holes on the wiring harness assembly. Then, take the 3D Print–PCB Housing + optopipe assembly, align it with the previous structure, and secure it in place using SCR1 and SCR2 at both ends of PCB1. If necessary, hold NUT1 and NUT2 in position from the top side while tightening SCR1 and SCR2 to ensure proper engagement, as shown in Fig. 7 − C. To confirm correct installation, press the button on the 3D2. If one of the three LEDs turns blue and another one starts blinking like shown in the Fig. 7 − D, the assembly has been successfully installed.
• If the test is successful, remember to press the button again to turn off the StimulHeat module.
• Finally, position the TED1 module and the wires respectively into the hole and the designated cable channels on 3D1.
• Before clipping the assembly onto a Valve Index controller, don’t forget to place the 3D3 over TED1 + HSK1 to ensure optimal contact between the hand and TED1.

• On 3D2, gently insert OPT1, OPT2 and OPT3 into the corresponding holes, as shown in Fig. 7 − A.

## 6 Operation instructions

To ensure the Stimulheat operates correctly, we have organised the instructions into two parts: Device Install / Uninstall on Valve Index controller and Device Connection Protocol.

### 6.1 Safety operation information

The project has been designed to ensure safe operation under normal usage conditions. Several safety measures have been implemented to minimize potential risks, particularly those related to overheating and electrical hazards. These measures are summarized below:

• All electronic components are enclosed within a protective housing under two bolted screw, which includes a single power button to control the entire device. This enclosure prevents direct user contact with electrical components and provides an additional layer of protection against electrical hazards.
• TED1 is capable of generating a maximum temperature differential of 60 °C between its two sides when operated at its maximum rated conditions (DC current of 2.2 A and voltage of 8.8 V). To ensure safe operation, the firmware integrated in PCB1 limits the TED1 current to a maximum of 0.6 A at a maximum voltage of 3.7 V, resulting in a temperature variation of approximately ± 7 °C at the hand-contact surface.
• THR1 and THR2 continuously monitor the temperature on both sides of TED1. A safety threshold of 42 °C is defined for THR1, which measures the temperature at the hand-contact surface, in order to prevent excessive heating and prevent discomfort or pain perception. This helps also to prevent the risk of burns.
• PCB1 integrates a LiPo battery charging management stage, including discharge monitoring and automatic charge termination when the battery reaches its full capacity, reducing the risk of overcharging.

### 6.2 StimulHeat Install/Uninstall on Valve Index controller

**Fig. 8:**
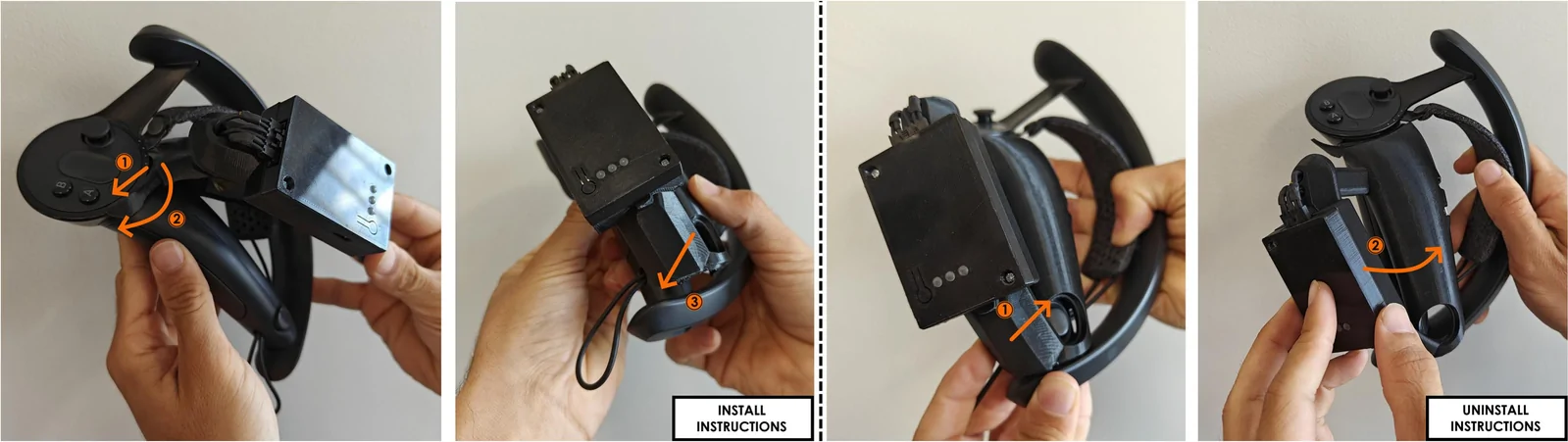
Operation instructions − Install-Uninstall StimulHeat on a Valve Index controller.

To install or uninstall StimulHeat on the Valve Index controller, take care to move the bottom part in order to not break the 3D print. In detailled, you can follow the sequence presented in Fig. 8.

### 6.3 StimulHeat Connection protocol − web App

StimulHeat communicates with its environment via the ISP1807 module, which integrates a Bluetooth Low Energy (BLE) chip. It uses the Google Protocol Buffers (ProtoBuf) protocol to encode and decode messages. The corresponding.proto file is included in the stimulheat isp1807 code.zip archive and can be used to integrate StimulHeat with any application that supports Bluetooth communication. To help you test your hardware, we have developed a local web application that allows you to control your StimulHeat device. Below, you’ll find a step-by-step tutorial on how to use the local web app.

**Fig. 9:**
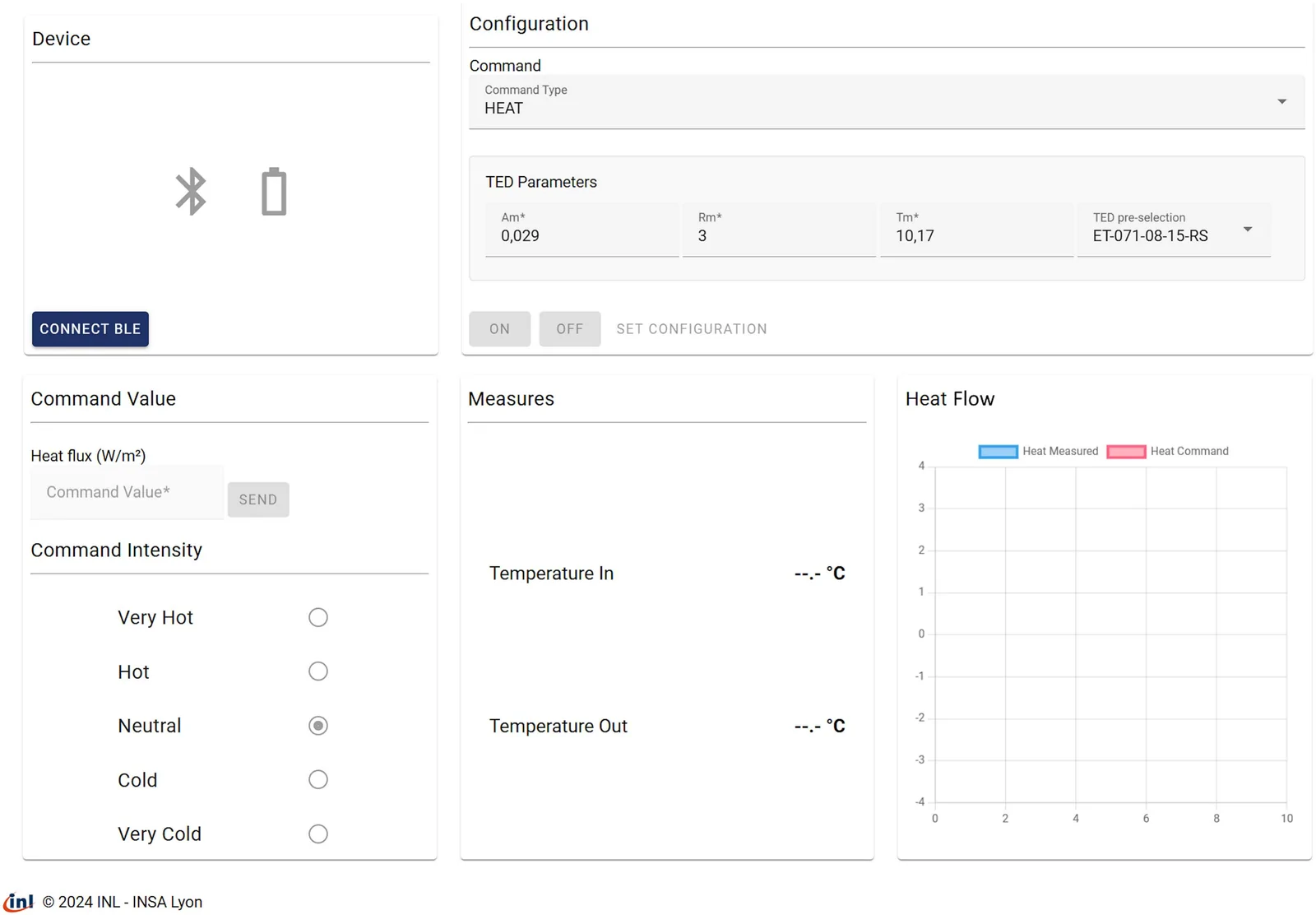
Operation instructions − Web App (screenshot).
• Put on your StimulHeat and click on ’CONNECT BLE’. You may find it in the list in the format ’StimulHeat XXXX’ with XXXX the serial number of your device. After selecting it, click on ’Connect’.
• You are now connected. To acess the command menu, you must enable the thermal control of the device by clicking on ’ON’. After that, you can change the value of the command as you want. To stop the use of the StimulHeat, click on ’OFF’

• First, download the ’stimulheat local web application.zip’ and extract all the files. Then, open with a web browser (not compatible with Firefox) the ’index.html’ file. You should find a web page similar to the one shown in Fig. 9.

**Important note:** At this stage, you may notice that the command response is inverted relative to the intended behavior. Do not worry—this simply indicates that the TED has been connected in reverse. To correct this, cut, strip, and swap the two relevant wires of the wiring harness before reconnecting them to the TED. This inversion will allow you to control the StimulHeat correctly via the web application.

### 6.4 StimulHeat connection protocol – Unity

**Fig. 10:**
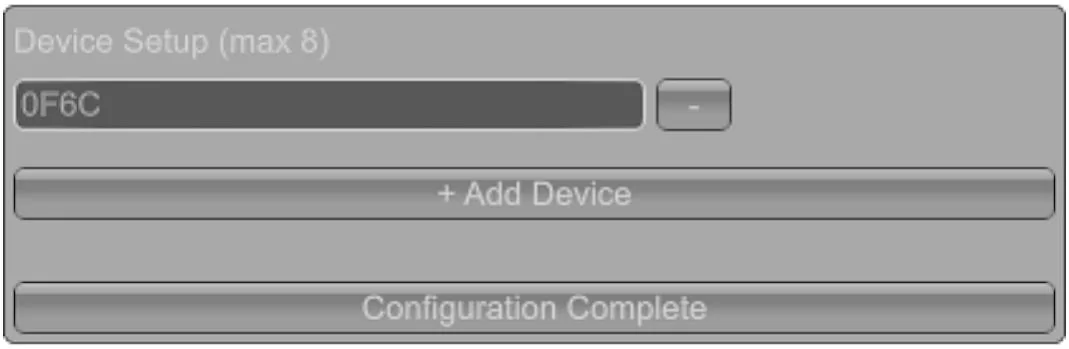
Configuration − Unity Desktop App (screenshot).

**Fig. 11:**
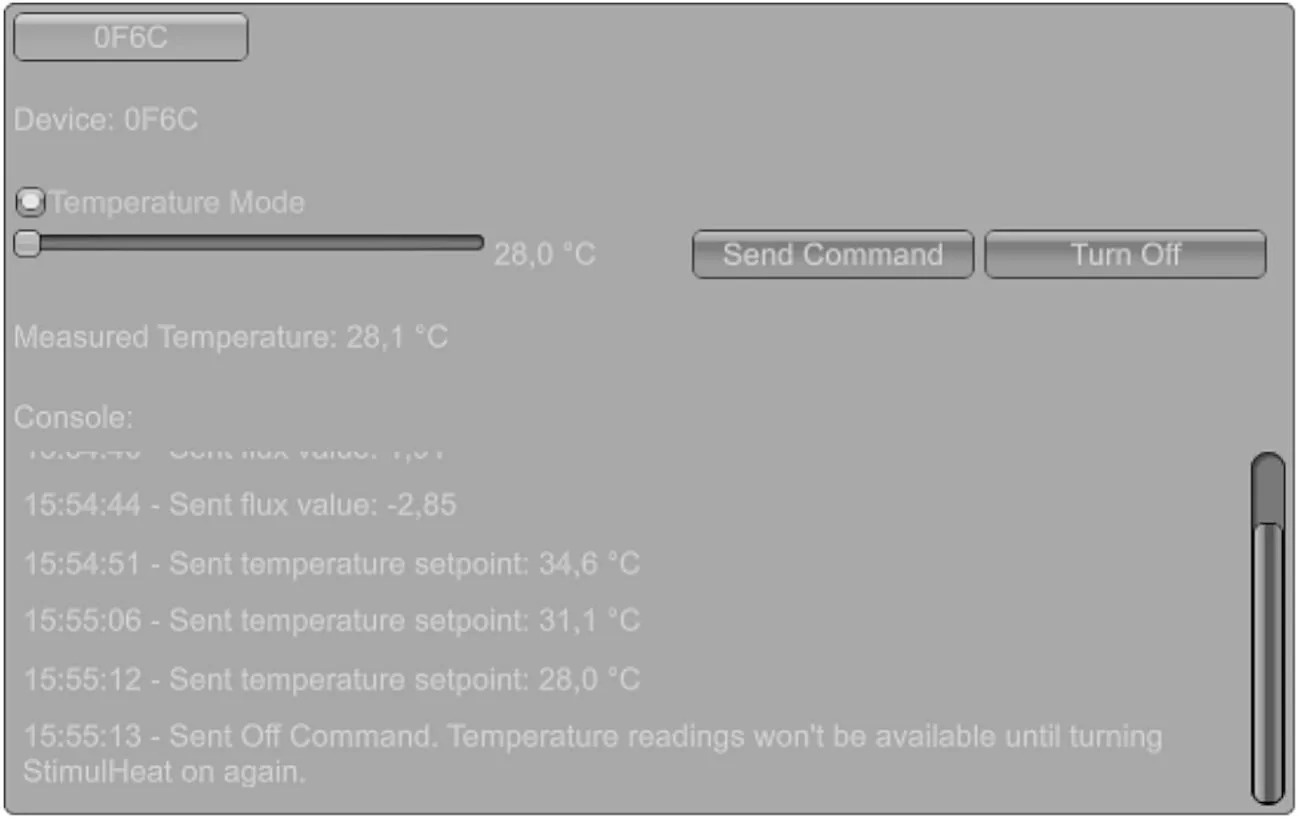
Control Panel − Unity Desktop App (screenshot).

To enable seamless integration of the StimulHeat device with Unity (https://unity.com/) based applications, we developed a dedicated package easily installable via Unity’s Package Manager, using the provided Git repository link. This package also bundles a desktop demo scene in the samples, they can be added directly in your Unity project from the Package Manager. After installation within your Unity Project, you can quickly configure and control the device by starting the bundled demo scene, located in *Assets/Samples/StimulHeat/0.0.1/Demo* (provided your computer is equipped with Bluetooth capabilities). If you don’t have Unity installed on your computer, you can also try out the demo application by launching the provided build. Once launched, the configuration interface (see Fig. 10) will prompt you to add your StimulHeat device’s public name (e.g., 0F6C), then click *Configuration Complete*. On the second screen (see Fig. 11), there is a panel for each of your connected StimulHeat device, from which you can switch the command type, i.e., Temperature or Flow, send commands and turn off the device.

## 7 Validation and characterization

To validate the functioning of the StimulHeat system, two complementary experiments were carried out. First, the custom-made current source was characterized by evaluating its voltage-to-current transfer function. Second, a full-scale validation experiment was performed to assess the overall hardware performance in terms of response time, overshoot and stability when utilised on a particular subject. Thereafter, a supplementary experiment was conducted to evaluate the hardware under its intended conditions of use, namely the generation of perceived warm and cool sensations. The experiment involves a number of participants (N = 16) who were requested to give feedback of their thermal sensation perceived and offer their impressions following the testing of StimulHeat in a VE via a survey. Although this second experiment does not fulfil all the objectives of a comprehensive hardware validation, it nevertheless provides valuable insights into the hardware's usability through evaluation by its intended end users. The two protocols followed for this user study were formerly approbated by the research ethics committee of Université de Lyon’s ComUE under approval number 2023-09-21-005.

### 7.1 Custom-made current source validation

For this first experiment, only the magnitude of the current delivered to the TED by the custom-designed current source was investigated. Based on the schematic shown in Fig. 13, the PIC16F17115 microcontroller, which controls the current source, was programmed to generate a DAC output voltage ($V_{\mathrm{DAC}}$) ranging from 0 to 0.6 V. In addition, the H-bridge was successively configured in both possible positions to reverse the polarity of the current supplied to the thermoelectric device (TED). The input voltages applied for each H-bridge configuration are listed below.

• $V_{\mathrm{DAC}}$ = [0,01 V; 0,06 V; 1,14 V; 0,21 V; 0,30 V; 0,37 V; 0,45 V; 0,51 V; 0,6 V]

**Fig. 12:**
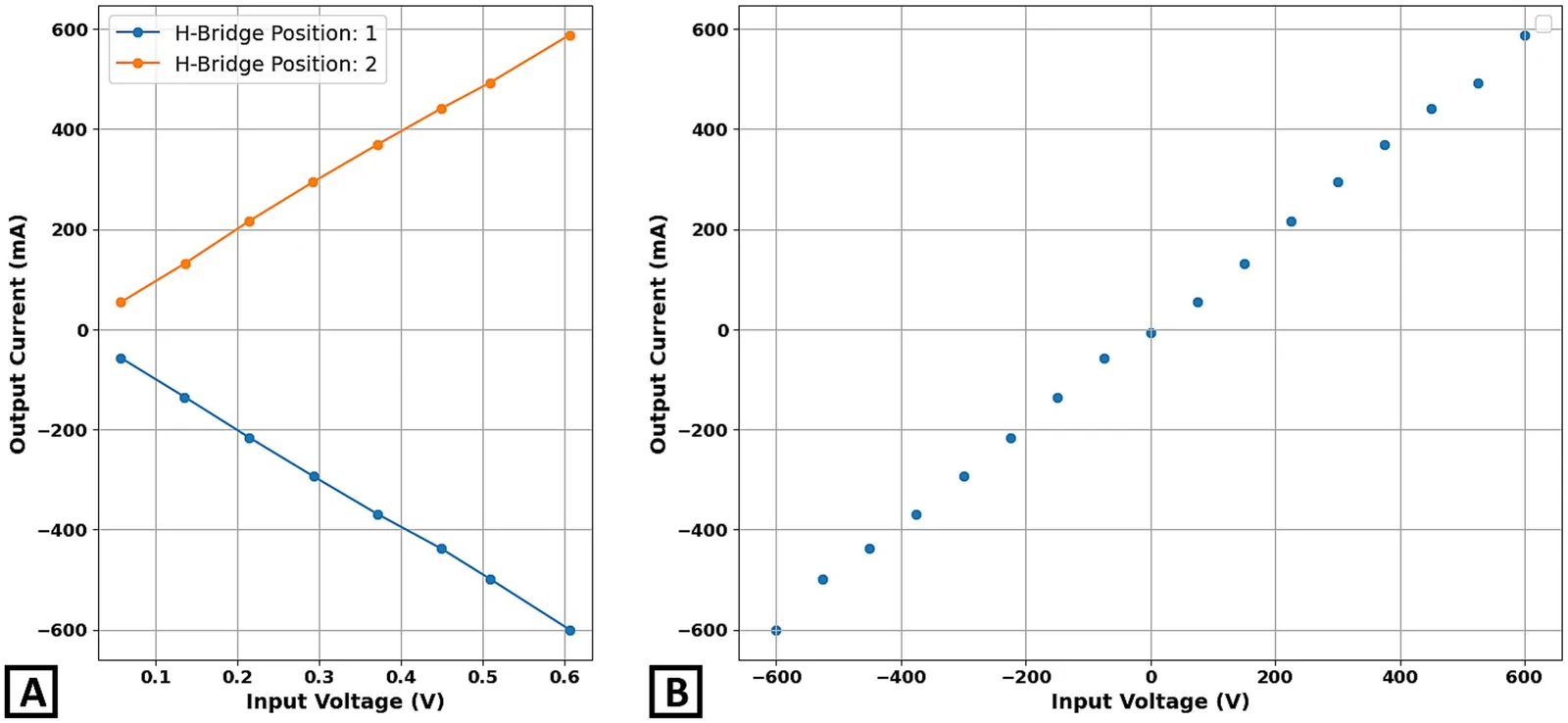
Characterization results of the custom-made current source. A − Voltage-to-current transfer function; B − Current setpoint-to-current transfer function.

As shown in the graph in Fig. 12 − A, the voltage-to-current transfer function is highly linear, demonstrating the proper operation of the adjustable bidirectional custom-made current source. Depending on the H-bridge configuration, the polarity of the current injected into the TED can be reversed. Consequently, by establishing a correspondence between the input voltage and the desired current setpoints (cf. Equation (3)), it is possible to derive the current control transfer characteristic, as illustrated in the graph in Fig. 12 − B. Specifically, when the current setpoint is positive, the H-bridge is configured in Position 1, and the corresponding input voltage is determined using equation (3), where:

- ${\mathrm{Input}}_{\mathrm{Voltage}}$: Input voltage applied to the custom-made current source (in Volt)
- ${\mathrm{Setpoint}}_{\mathrm{Current}}$: Desired output current to be injected into the TED (in Ampere)

**Fig. 13:**
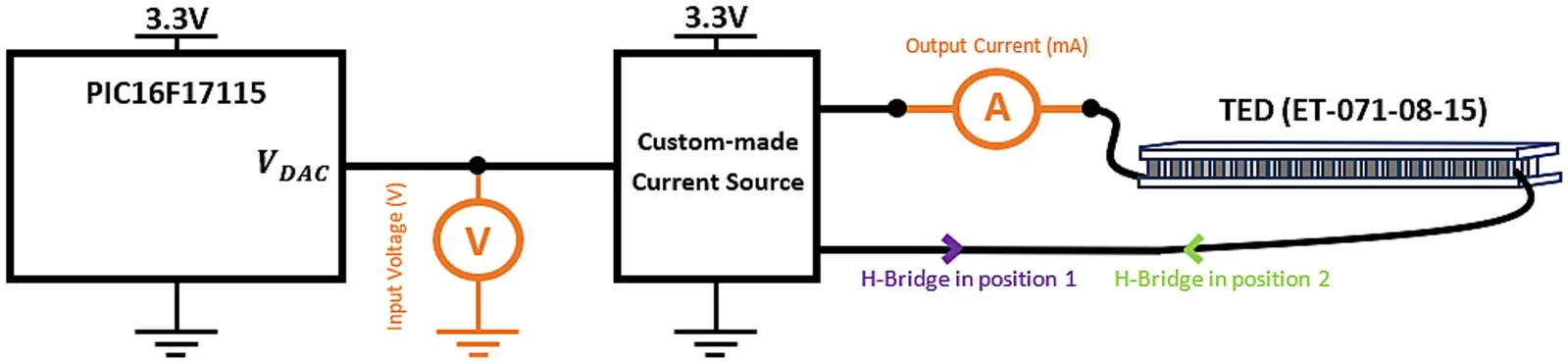
Schematic of the custom-made current source validation test.

Conversely, when the current setpoint is negative, the H-bridge is switched to Position 2, and the corresponding input voltage is calculated using the same equation.

(3) $${\mathrm{Input}}_{\mathrm{Voltage}}=\frac{\left|{\mathrm{Setpoint}}_{\mathrm{Current}}\right|}{980}$$

### 7.2 StimulHeat technical characterization

#### 7.2.1 Materials and methods

The primary objective of the present study was to characterise StimulHeat in common-use conditions in VR. In order to achieve this goal, a single protocol was developed, which involved one healthy participant (male, aged 25). He was tasked to maintain the Valve Index controller with StimulHeat integration during a period of several technical trials. A total of 16 distinct thermal stimuli, 8 heat flow setpoints and 8 temperature setpoints, were administered in accordance with the subsequent protocol: firstly, a 5-second waiting period was maintained (0 W or 35 °C, depending on whether the control was set to heat or temperature), followed by a 5-second thermal stimulus. This stimulus duration was chosen to assess the performance of the proposed system in reproducing short-duration thermal contact sensations. It guaranteed the skin in contact with the TED to be returned to its initial state (T_skin_ = 35 °C) before conducting the subsequent stimulation. This temperature measurement was obtained under the specific environmental conditions prevailing on the day of the experiment, namely during the summer of 2025. The range of skin temperatures generally associated with a neutral thermal sensation is between 30 °C and 36 °C, according to the climatic context [24]. The lists of commands administered are detailed hereafter:

• Heat flow: [- 4 W; - 3 W; - 2 W; - 1 W; 1 W; 2 W; 3 W; 4 W]
• Temperature: [28 °C; 30 °C; 32 °C; 34 °C; 36 °C; 38 °C; 40 °C; 42 °C]

**Fig. 14:**
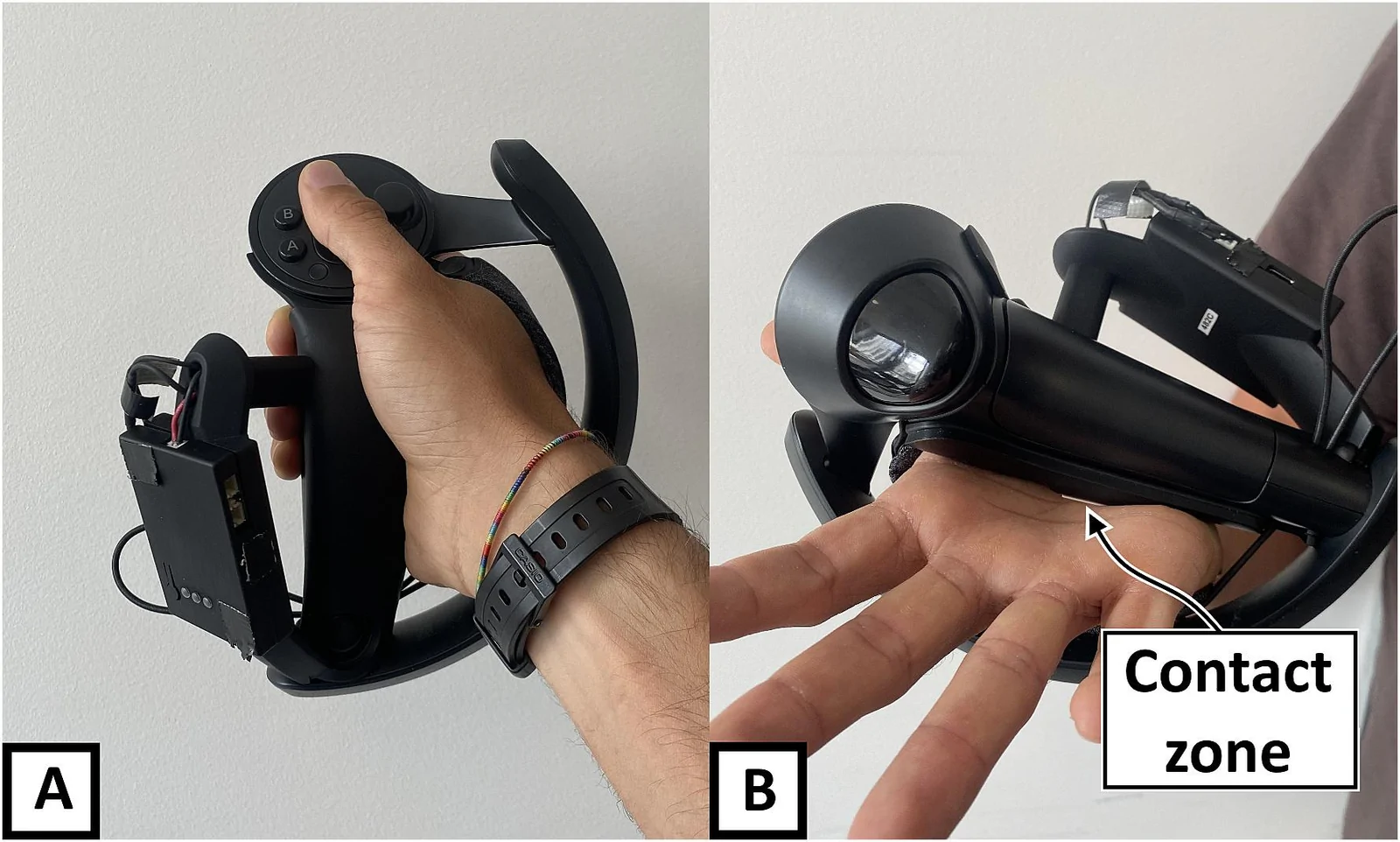
Technical Characterisation − Protocol Picture. A − Overview of the characterization protocol; B − Close-up of the contact surface stimulated during the characterization protocol.

During the sequences, the temperature of the two thermistors was measured. The equation (2) was utilised to deduce the heat flow present in the TED, facilitating the comparison between the setpoint in heat flow and the real heat flow. Additionally, the temperature of the skin at the contact point between the palm and the TED was determined, permitting a comparison between the setpoint in temperature and actual temperature (see Fig. 14).

The objective of this full-scale validation test is firstly to compare two methods of controlling the StimulHeat device and secondly to determine the advantages and disadvantages of each. This characterisation is made in use condition when StimulHeat is wear by the subject, but no consideration has been given to the thermal feelings perceived. The analysis of this feedback is presented in the supplementary study, which assesses whether the device can provide hot and cold sensations in response to a given command.

#### 7.2.2 Results and discussion

**Fig. 15:**
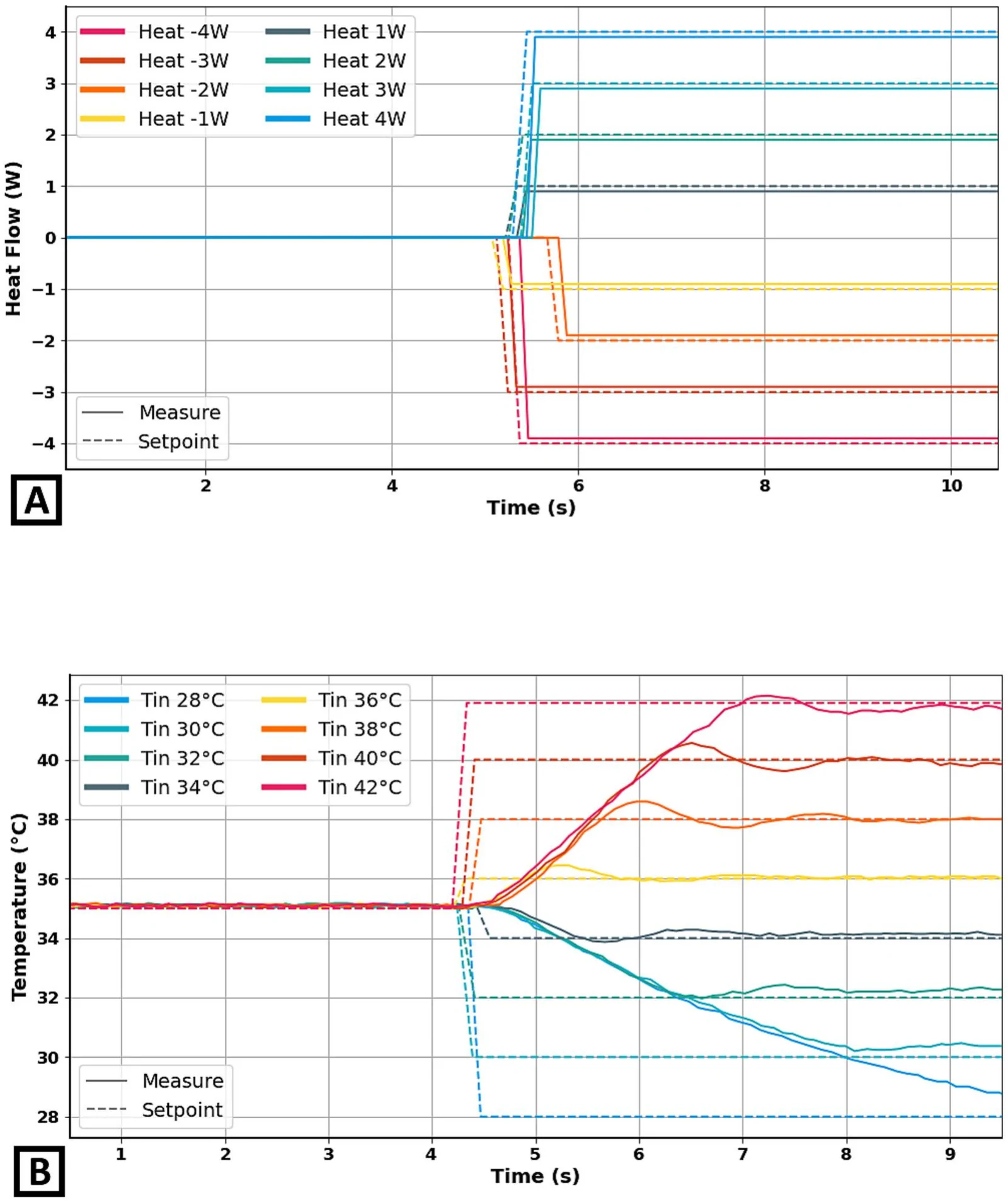
Technical Characterisation − Global Results. A − Results obtained under the heat flow setpoint condition; B − Results obtained under the temperature setpoint condition.

Table 3 highlights a clear difference between heat flow control and temperature control in dynamic performance showed in Fig. 15. The heat flow control exhibited consistently low tracking errors, with an RMSE of 0.1 W across all tested heat flow setpoints (- 4 W to + 4 W). Moreover, it showed no overshoot (0 %) under any operating condition and reached steady state within 0.1 s for every test, demonstrating excellent accuracy, stability, and responsiveness.

**Table 3:** Quantitative performance metrics for the comparison of the two StimulHeat control approaches based on heat flow and temperature regulation.

Command Type	Heat Flow			Temperature		
	*Setpoint*			*Setpoint*		
Root Mean Squared Error (RMSE) Calculated over the steady-state region, corresponding to the last second of stimulation (from 8 to 9 s).		- 4 W	0,1 W		42 °C	0.23 °C
		- 3 W	0,1 W		40 °C	0.07 °C
		- 2 W	0,1 W		38 °C	0.07 °C
		- 1 W	0,1 W		36 °C	0.06 °C
		1 W	0,1 W		34 °C	0.14 °C
		2 W	0,1 W		32 °C	0.21 °C
		3 W	0,1 W		30 °C	0.32 °C
		4 W	0,1 W		28 °C	1.57 °C

Overshoot		- 4 W	0 %		42 °C	0,60 %
		- 3 W	0 %		40 °C	1,40 %
		- 2 W	0 %		38 °C	1,55 %
		- 1 W	0 %		36 °C	1,25 %
		1 W	0 %		34 °C	0,40 %
		2 W	0 %		32 °C	0 %
		3 W	0 %		30 °C	0 %
		4 W	0 %		28 °C	0 %

Settling Time − ± 2,5 %		- 4 W	0,1 s		42 °C	2,18 s
		- 3 W	0,1 s		40 °C	1,41 s
		- 2 W	0,1 s		38 °C	0,90 s
		- 1 W	0,1 s		36 °C	0,21 s
		1 W	0,1 s		34 °C	0,29 s
		2 W	0,1 s		32 °C	1,41 s
		3 W	0,1 s		30 °C	2,78 s
		4 W	0,1 s		28 °C	4,02 s

In contrast, the temperature control mode showed greater variability depending on the target temperature. The RMSE remained relatively low between 30 °C and 42 °C (0.06 – 0.32 °C), but increased significantly at 28 °C, reaching 1.57 °C, indicating reduced tracking accuracy at the lowest setpoint. A small overshoot was observed at higher temperature setpoints (up to 1.55 % at 38 °C), while no overshoot occurred at 32 °C and below. Similarly, the settling time strongly depended on the operating point, ranging from 0.21 s (36 °C) to 4.02 s (28 °C), with slower responses at lower temperature setpoints.

This allows us to conclude that heat control responds more quickly than temperature control. This result is expected, since a TED’s heat flow is directly linked to the value of the current injected into it. In contrast, the temperature measured at the point of contact with the skin is not directly correlated with the injected current. This enhances the accuracy and robustness of the experimental conditions in heat flow-based control in comparison to temperature-based control, as it is independent of the PID parameter values.

To resume, the capabilities are the following:

• Heat flow control strategy provides a highly precise tracking of the setpoint, with low steady-state errors (RMSE = 0.1 W) over the entire tested range (±1 W to ± 4 W).
• Heat flow control achieves also a settling time of approximately 0.1 s for all tested setpoints, demonstrating a rapid response of the system.
• No overshoot was observed during heat flow regulation, indicating a stable control behavior even for positive and negative heat flow commands.
• Regarding the temperature control strategy, it maintains a low overshoot (<1.6 %) and achieves the best performance around the physiological temperature range (34 – 38 °C).

To summarize the limitations:

• Temperature control exhibits slower dynamics, with settling times increasing up to several seconds for extreme temperature setpoints due to the intrinsic thermal response of the system caused by the saturation of the command.
• Temperature regulation performance decreases for low (28 – 30 °C) and high (40 – 42 °C) setpoints, resulting in larger tracking errors (RMSE up to 1.57 °C).
• Accuracy of the temperature control strongly depends on the operating point, unlike the heat flow regulation which remains almost constant over the tested range.
• The present system was designed to reproduce short-duration thermal contact sensations (approximately 5 s). Consequently, the reported performance is limited to this specific use case, and the experimental protocol would need to be adapted and repeated to assess the system's performance during longer thermal stimulation periods.

### 7.3 Supplementary protocol: VR user assessment

#### 7.3.1 Materials and methods

**Fig. 16:**
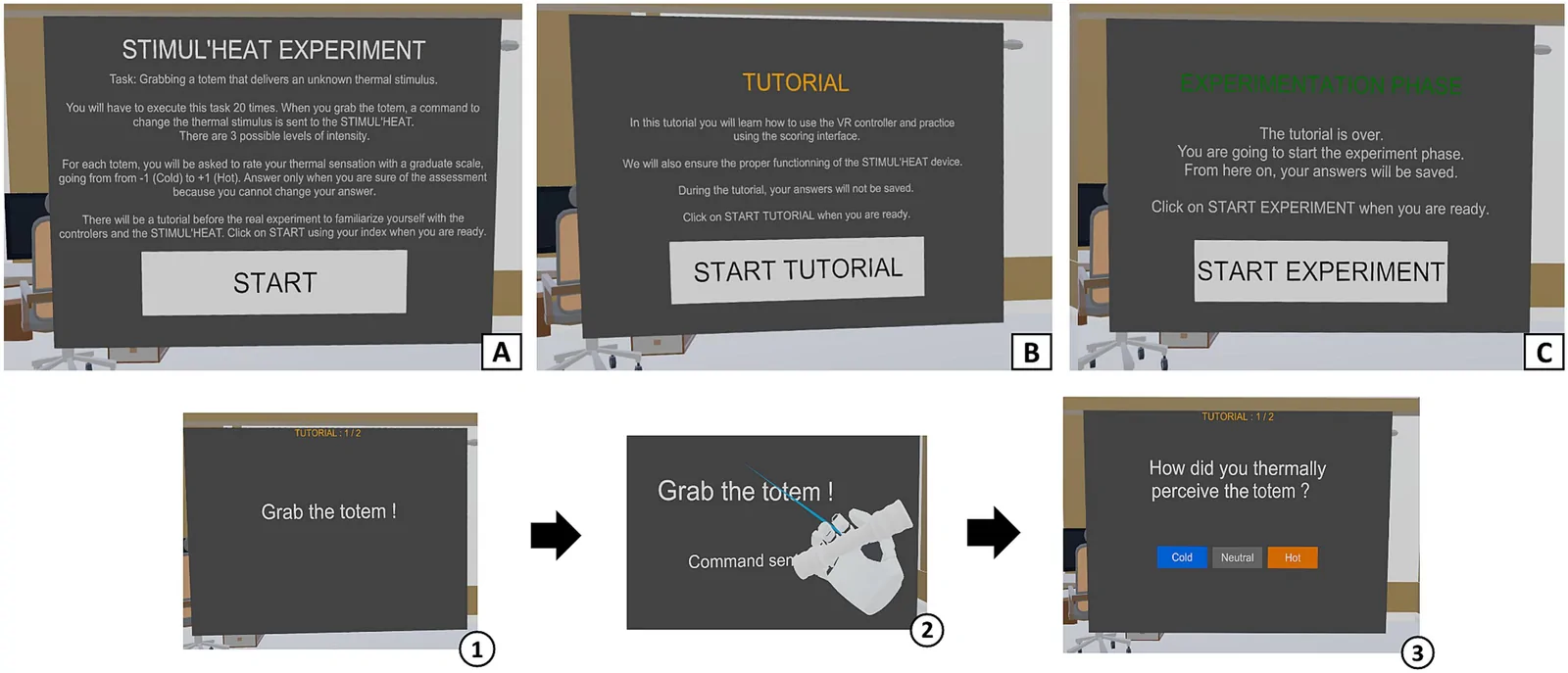
User experience – Protocol. Top: VE interface shown to participants at each stage — A: Presentation panel at experiment start; B: Tutorial panel after clicking”Start”; C: Experimentation panel after tutorial completion. Bottom: Evaluation protocol for each totem — 1: After a 5-second delay, the totem appears with a prompt to grab it; 2: Upon grabbing, a 5-second heat stimulus is delivered; 3: After a 3-second wait, participants rate the thermal sensation as COLD, NEUTRAL or HOT.

The goal of the second study was to validate the device in a VR setup use case. In this investigation, a novel protocol was developed, based on the subject’s thermal sensation and the study of StimulHeat’s user-friendliness and intrusiveness. Its objective is to assess the device's ability to reliably elicit the commanded sensations of warmth and cold, outcomes that cannot be objectively quantified without involving human participants. Therefore, the study was conducted with 16 participants (N = 16), ranging in age from 20 to 39, including both male and female subjects (H = 9; F = 7). The participants were immersed in a VE for a period of approximately ten minutes and requested to grasp a blank totem on 20 occasions (2 for the tutorial and 18 for the experiment). Following a preliminary briefing, during which the explanation and consent document were given, the participants placed the VR headset and entered the VE. The visual representation of the VR scenario is illustrated in Fig. 16. With each act of”picking up a totem” by the user, a heat command was applied to the StimulHeat controller on the hand which grasped the totem (either left or right, or twice if the user grabs the totem with both hands). The decision was taken to utilise the heat flow command due to its enhanced stability and reactivity in comparison to the temperature command. The command was either HOT (- 2 W), NEUTRAL (0 W) or COLD (2 W). The thermal stimulation was administered for a duration of 5 s. In this period, the participants were requested to indicate the precise moment when they began to perceive the thermal stimuli by pressing a button on the Valve Index controller. This data contributes to our understanding of reaction time, which encompasses both the device’s response time and the time required to perceive a sensory stimulus. At the end of the thermal stimulation, the totem then disappeared, and the participants had three seconds to consider the most accurate description of the perceived stimulation. They were then invited to select if the stimulus was perceived as either: HOT, NEUTRAL or COLD. The sequence of stimuli was administered identically to all participants and was as follows:

• Heat flow: [2 W; 0 W; - 2 W; 2 W; - 2 W; 2 W; 0 W; - 2 W; 0 W; - 2 W; 2 W; 0 W; 2 W; - 2 W; 2 W; 0 W; - 2 W; 0 W]

**Fig. 17:**
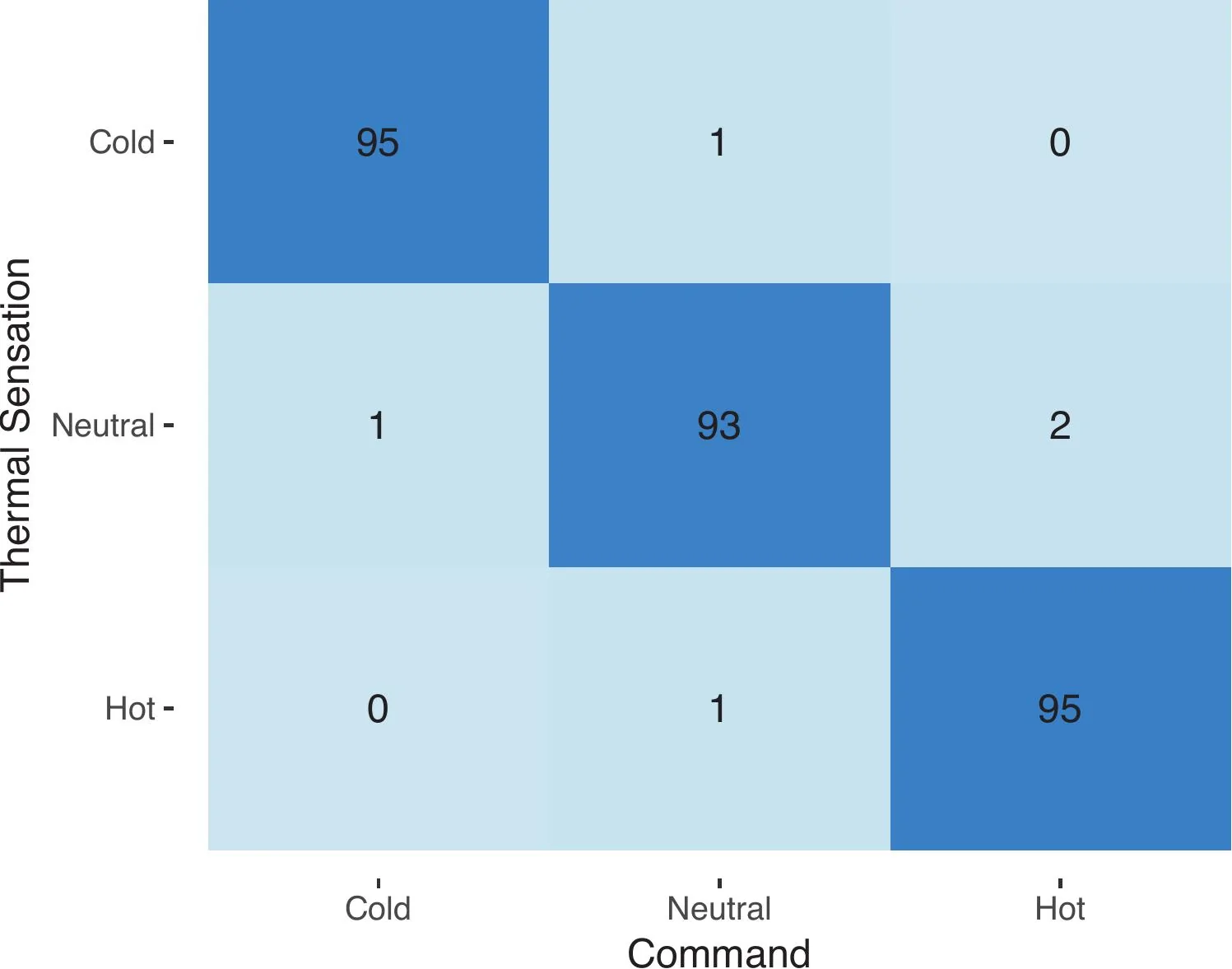
User validation – Thermal perception confusion matrix.

Following this, all the answers were collected and compared using a correlation matrix presented in Fig. 17, which plots the relationship between the command given and the thermal sensation perceived and communicated by the participants.

Comfort of use was also evaluated. To assess this, a survey with three affirmations was administered to each participant. We used a scale ranging from 1 to 7, where 1 denotes ’Strongly Disagree’ and 7 denotes ’Strongly Agree’ with the corresponding affirmation. In order to ensure an unbiased experience in relation to the use of VR, the panel of participants was composed of three different groups: one with people who have never used VR (Group ”None”), one with participants who have already used VR at least once (Group ”Beginner”), and one with participants who are familiar with the technology and have used it more than once (Group ”Intermediate”). The number of participants in each group were N_None_ = 5, N_Beginner_ = 6, and N_Intermediate_ = 5. The three 18 affirmations of the survey are presented hereafter:

• I experienced thermal discomfort on my hand while using the StimulHeat device during the experiment.
• I experienced discomfort other than thermal while using the StimulHeat device during the experiment.
• The controller felt too large for my hands and made it difficult to grasp objects.

**Fig. 18:**
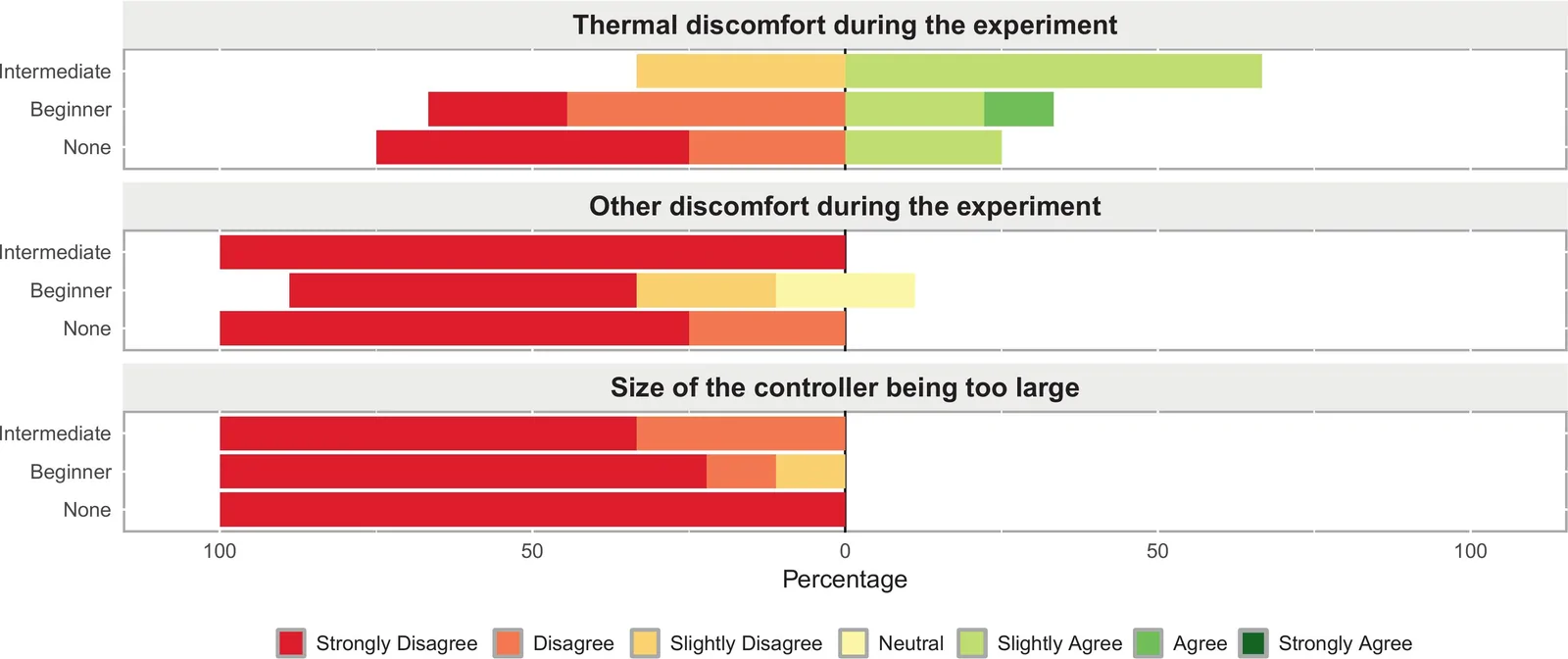
User validation – Survey results group by level of VR experience.

The results obtained from the experiment are presented in Fig. 18, thus enabling a comparative analysis of the responses to the affirmations according to each group.

### 7.4 Results and discussion

Regarding the correlation matrix in the Fig. 17 which establishes a relationship between the perceived thermal sensation and the command, StimulHeat demonstrates a high degree of precision in regard to these three heat commands. Furthermore, when analysing the data exclusively in terms of failed predictions, it is notable that no instance of Hot Command has been experienced as Cold sensation, and vice versa. It can be added that in instances where the device is utilised to induce either a hot or a cold thermal sensation, there were two cases on 192 whose the command failed to generate such a sensation. Three cases on 96 were registered in which a thermal sensation was perceived, despite the absence of any command injection in the TED.

Next, regarding the boxplots in Fig. 18, several deductions can be made. Regarding the initial statement on thermal discomfort, it appears that the intermediate group has encountered a higher level of discomfort in comparison to the beginner and none groups. This could possibly be explained by the fact that for the two groups mentioned above, this experience stimulates their curiosity and the discovery of the technology and perhaps inhibits their ability to feel the surrounding context. Nevertheless, the identified thermal discomfort remains low even for the Intermediate group’s VR experience. Turning to the boxplot relating to the controller size perceived as excessive for the hand, it can be deduced from the responses that the incorporation of StimulHeat into the controller does not compromise the user experience or comfort during a VR session, as none of the participants from all groups expressed any opinion in favour of this statement. These results are supported by the boxplot associated with the second affirmation, which concerns the discomfort experienced during VR (virtual reality) sessions, different from thermal discomfort. The results of the study indicated that the majority of participants did not agree with the proposal, and thus that the integration design of the StimulHeat did not cause discomfort.

### 7.5 Capabilities and limitations

Based on the validation results, the main hardware capabilities and limitations of the proposed system can be summarized as follows.

Capabilities:

• The system can generate a temperature variation of ± 7 °C and maintain a heat flow of ± 4 W over a localized skin contact area.
• The system provides a short response time of less than 1 s in heat flow control mode and a few seconds in temperature control mode.
• The system is designed for the Valve Index controller and can be adapted to other VR controllers by redesigning the external shell.
• The system can be remotely controlled via Bluetooth Low Energy (BLE) communication.
• The system is powered by an 800 mAh, 3.7 V LiPo rechargeable battery and consumes up to 2.22 W. Under typical operating conditions, the average power consumption is approximately 1.48 W (0.4 A × 3.7 V), providing an autonomy of about 2 h, which is suitable for typical VR sessions.

Limitations:

• The system cannot maintain VERY COLD stimulation for extended periods (>1 min) because of the progressive loss of cooling efficiency of the thermoelectric device (TED).
• Under prolonged extreme cooling conditions, the TED may gradually lose its cooling capacity, resulting in a degradation of the intended thermal stimulus. This capacity strongly depends on the efficiency of the heat dissipation solution, which remains challenging to optimize in a compact wearable form factor.
• More advanced cooling solutions could improve long-term thermal stability and longer-duration thermal contact sensation [25] but would increase energy consumption, device weight, and system intrusiveness.

## Ethics statements

As the study incorporates human subjects, consent was obtained from the volunteers who used StimulHeat via a brief informational session prior to the experiment and a consent agreement. This study was approved by the research ethics committee of Université de Lyon’s ComUE, under approval number 2023-09-21-005.

## CRediT authorship contribution statement

**Matthieu Mesnage:** Writing – review & editing, Writing – original draft, Validation, Software, Methodology, Conceptualization. **Sophie Villenave:** Writing – review & editing, Visualization, Validation, Software, Formal analysis, Data curation. **Bertrand Massot:** Writing – review & editing, Validation, Supervision, Software, Conceptualization. **Matthieu Blanchard:** Writing – review & editing, Validation, Software. **Pierre Raimbaud:** Writing – review & editing, Supervision. **Guillaume Lavoué:** Writing – review & editing, Validation, Supervision, Software. **Claudine Gehin:** Supervision.

## Declaration of competing interest

The authors declare that they have no known competing financial interests or personal relationships that could have appeared to influence the work reported in this paper.
